# Hydrogel-Based Sensors: Compositions, Fabrication, Sensing Mechanism, and Applications

**DOI:** 10.3390/polym18121455

**Published:** 2026-06-10

**Authors:** Hassanain Ali, Xiao-Feng Sun, Zeesham Ali, Ran Sun, Sihai Hu

**Affiliations:** 1 School of Chemistry and Chemical Engineering, Northwestern Polytechnical University, Xi’an 710129, China; alihassanain@mail.nwpu.edu.cn (H.A.); husihai@nwpu.edu.cn (S.H.); 2 Shenzhen Research Institute of Northwestern Polytechnical University, Shenzhen 518057, China; 3 College of New Materials and Nanotechnolgies, National University of Science and Technology MISIS, Moscow 119049, Russia

**Keywords:** hydrogel-based sensors, natural polymers, synthetic polymers, sensing mechanisms, biomedical and environmental monitoring

## Abstract

Hydrogel-based sensors have emerged as transformative soft-sensing platforms, featuring tissue-matched compliance, high water content, stimuli responsiveness, and chemical tunability, properties which are unachievable with conventional rigid sensors. Despite substantial advances, the existing reviews focus on individual polymer categories, discrete transduction mechanisms, or targeted standalone applications, failing to establish an integrated pipeline from material design to final sensing performance. This review fills these crucial gaps by systematically correlating polymer chemistry, crosslinking tactics, and fabrication protocols with the selection of transduction mechanisms and resultant sensing performance across biomedical and environmental fields. We conduct a critical assessment of natural and synthetic polymers together with chemical, physical, and hybrid composite crosslinking methodologies. Multiple sensing modalities, including piezoresistive, capacitive, thermogalvanic, electrochemical, colorimetric, ratiometric fluorescence, and piezoionic sensing are elaborated alongside representative quantitative performance parameters. Emerging platforms, including self-powered thermogalvanic sensors, SERS-integrated biosensors, and MXene/MOF composites, are highlighted as underexplored frontiers. In addition, persistent bottlenecks including dehydration-derived signal drift, inferior long-term operational stability, unsatisfactory target selectivity, and obstacles toward large-scale manufacturability are rigorously analyzed. Ultimately, this review constructs a holistic unified framework bridging polymer molecular design, fabrication engineering, signal transduction, and practical end-use applications, laying a clear developmental roadmap for next-generation flexible and smart hydrogel-based sensing systems.

## 1. Introduction

**Figure 1:**
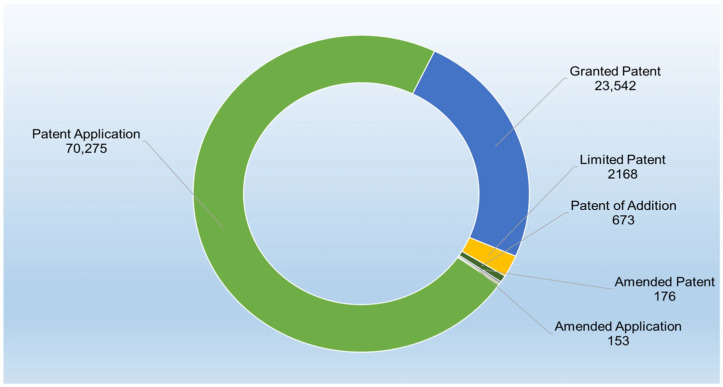
Different types of patent documents and their corresponding counts [24]. Copyrights: image has been taken and reproduced from © 2024, MDPI.

Hydrogels are three-dimensional crosslinked polymer networks with high water content which confer biocompatibility, mechanical flexibility, and tunable responsiveness to external stimuli [1]. Currently, the global hydrogel market is valued at approximately $23.4 billion in 2023. This value is expected to be around $45.7 billion by 2033, with a compound annual growth rate (CAGR) of 6.9% from 2024 to 2033 [2]. The rapid growth in the hydrogel market is attributed to its diverse fields across water treatment [3], biochemical analysis [4,5,6,7], tissue engineering [8], drug delivery [9,10], agricultural applications [11,12,13], soft robotics [14], and wearable sensors [15]. Within these domains, hydrogels have found specific utility in wound treatment [16], responsive smart window [17,18], soft actuators [19,20], shape deformations [21], e-skin, communication aids, electromagnetic protection [22], clinical diagnostics, regenerative medicine, skin regeneration, and implantable devices [23]. The growing importance of hydrogels is further reflected in patenting activity. As of 15 September 2024, a total of 96,987 patent documents have been published, encompassing various types such as patent applications, granted patents, and amended patents. This robust patent landscape underscores the technological maturity and expanding innovation in hydrogel-based materials (Figure 1) [24]. Thus, it is revealed that hydrogels have great importance and application. Moreover, the current development in the design of smart/stimuli-responsive hydrogels boosts their prospective to function as intelligent and sophisticated systems in different biomedical applications [25]. Also, stimuli-responsive hydrogels have emerged as a research hotspot in the renewable energy field by their successful integration into energy storage, energy conversion, and intelligent energy management systems, demonstrating their versatility as a platform material extending across the broader landscape of advanced functional technologies [26].

Hydrogels exhibit an extraordinarily broad range of applications, such as adsorbents, electrolytes, electrode materials, and carriers for drug delivery. Among these varied uses, their implementation as sensing platforms has evolved into one of the most scientifically meaningful and rapidly expanding research fields. Hydrogel-based sensors have recently attracted considerable attention owing to the intrinsic sensing properties of hydrogels, particularly their stimuli-responsiveness, which is inherently aligned with the demands of modern sensing across diverse domains. A distinctive advantage of hydrogels is that they can be readily functionalized for the desired sensing target [27,28]. For instance, Tang et al. (2020) fabricated a cost-effective and reproducible glucose sensor using TEMPO-oxidized cellulose nanocrystals (CNCs) as the supporting matrix, and the sensor exhibited a linear detection range of 0.1–2 mM, a sensitivity of 5.7 ± 0.3 µA cm^−2^·mM^−1^, and a limit of detection of 0.004 mM, and it retained 92.3% of its initial response after 30 consecutive measurements and demonstrated a shelf life of one month [29]. The sensor was successfully applied to monitor glucose consumption in fibroblast cell cultures [29,30], while Wang et al. (2025) demonstrated a dual functionalization cellulose nanofiller incorporated into a polyacrylic acid (PAA) hydrogel sensor capable of simultaneously enhancing mechanical properties (stress–strain from 2.91 MPa and 321.23% to 4.69 MPa and 512.73%) and electrical conductivity (from 0.12 to 0.58 Siemens per meter (S/m)), achieving a 4.7-fold increase in conductivity. This inherent functional versatility has driven interest in understanding how hydrogel-based sensors compare to conventional sensing platforms [31]. This inherent functional versatility has driven interest in understanding how hydrogel-based sensors compare to conventional sensing platforms. While conventional sensors typically rely on rigid, elastic substrates and physical transduction mechanisms, hydrogel-based sensors exploit their soft, hydrated nature and chemical responsiveness to achieve distinct sensing modalities [32].

Conventional electronic sensors rely on rigid synthetic materials and suffer from unstable conductivity under dynamic conditions. By contrast, hydrogel-based ionic sensors transport ions through polymer networks to achieve conformable skin contact and high-fidelity biological interfacing [33]. Recent advances in biomass-based dual-network hydrogels have overcome bottlenecks in mechanical strength and adhesion, enabling multifunctional platforms that integrate flexible sensing and rapid hemostasis. Similarly, Oh et al. (2024) discussed that the conventional rigid sensors (modulus ~1–42 Giga pascal (GPa)) cause inflammation and tissue damage due to mechanical mismatch. Hydrogel-based sensors soften post-implantation from ~50 MPa to ~16.5 Kilopascal (kPa) (a 3000× reduction), reducing glial scarring and improving conformal contact. However, hydrogels alone cannot penetrate tissue (bending stiffness 0.42 N/m) without a temporary rigid support (e.g., silk fibroin with initial modulus 38.7 MPa). The optimal solution is a softening device that starts rigid (e.g., 2380 MPa) and softens in vivo (to ~550 MPa or lower) via temperature or fluid absorption [34]. In rigid bioelectronic devices and soft biological tissue certain challenges of mechanical mismatch arise, especially in long-term applications, including tissue degeneration, signal interference, poor adhesion noise level, and device stability; however, hydrogel-based sensors combine tissue-like mechanical properties (kPa modulus, >70% water content) with bioadhesive, self-healing, and permeable functionalities. This enables low-impedance, high-SNR signal transmission with minimal inflammation, tissue degeneration, and device instability, making them ideal for long-term wearable and implantable bioelectronic applications [32]. Furthermore, conventional wearable sensors rely on indirect signal transduction through external rigid electronic components, limiting their miniaturization and integration to dynamic biological environments [35]. Hydrogel-based sensors overcome these limitations due to their intrinsic properties and compatibility. Combined with the properties, hydrogel-based sensors are mechanistically superior and biologically integrated alternative to conventional rigid sensing technologies across biomedical and environmental monitoring domains [36]. Compared to conventional rigid sensors, hydrogel-based sensors offer several fundamental advantages rooted in their material architecture.

Conventional sensors rely on rigid substrates with moduli in the GPa range, causing mechanical mismatch with biological tissues (modulus ~1–100 kPa) that leads to inflammation, signal interference, and device instability during long-term use [32,34]. By contrast, hydrogel-based sensors operate at tissue-matched moduli below 10 kPa, achieve water contents exceeding 70%, and enable conformal skin contact with impedances orders of magnitude lower than rigid electrodes [32,37]. In terms of sensing performance, conventional sensors typically offer higher absolute conductivity with metallic electrodes reaching 10^6^ S/m but lack stimuli-responsiveness, self-healing, and chemical tunability. Hydrogel-based ionic sensors achieve a conductivity of 0.2–1850 S/m [37,38] which, while lower than metals, is sufficient for high-fidelity biosignal acquisition, and uniquely combines mechanical compliance with simultaneous chemical, thermal, and mechanical sensing modalities within a single material platform. Furthermore, hydrogels can be functionalized with enzymes, aptamers, and optical probes to enable direct analyte-responsive transduction without external reagents, a capability absent from conventional rigid sensing architectures [39,40].

Hydrogel-based sensors, unlike passive transducers, exploit material-level responses such as swelling, conductivity changes, and optical shifts to directly convert stimuli into measurable signals without rigid electronic intermediaries [38,41]. Depending on their composition and functionalization, hydrogel sensors can sense [42] pH [43,44], temperature [45,46], humidity [47], mechanical strain [48], and biomolecules [39] from wearable health monitoring devices [49] to environmental sensing platforms [50,51]. Moreover, due to their versatility and compatibility, hydrogel-based sensors are employed across biological, non-biological [52], and environmental applications [53]. In medicine, hydrogel-based sensors are being developed for electrophysiological monitoring [54], interstitial fluid analysis [55], wound assessment [56], and minimally invasive biosensing wherein hydrogel microneedle arrays or transdermal patches access interstitial fluid through superficial skin penetration, enabling continuous biomarker monitoring without venipuncture [57]. In broader sensing contexts, these devices can also detect various analytes, including drugs, heavy metals, and environmental contaminants. Particularly, notable progress has been achieved in wearable systems, including ultrathin hydrogel electronic tattoo sensors [58]. These advances highlight the capacity of hydrogel materials to support long-term, skin-conformal, and high-performance sensing [59]. Recent studies reported a hydrogel-based electronic tattoo sensor with thicknesses as low as 20 μm, a Young’s modulus of 31 kPa, and operational lifetimes exceeding six months demonstrating how materials engineering is translating laboratory concepts into practical sensor technologies [58,59,60].

Despite these developments, a number of obstacles still need to be overcome before hydrogel-based sensors may be widely used in clinical and industrial settings. Real-world performance is still constrained by long-term water retention, interfacial stability, antifouling behavior, mechanical robustness, and scalable production. Hydrogel-based sensors are considered promising materials for flexible health monitoring [38]. In the next generation of wearable electronics and human interactive systems, hydrogel-based flexible sensors proved to be transformative and innovative. These hydrogels containing more than 50 wt% (weight percentage) water, can show cell viability above 80%, achieve very soft modulus values below 10 kPa, allow the thermal tuning of LCST (Lower Critical Solution Temperature) from 34.3 °C to 29.5 °C, reach conductivity as high as 1850 S/m, stretch up to 2800%, recover 93% of conductivity within 1 h after self-healing, and respond rapidly in sensing applications, with a temperature sensitivity of 3.5%/°C and a glucose response time of 3 s [37]. Moreover, hydrogel-based sensors open up possibilities for real-time, targeted neurochemical monitoring in intricate and dynamic biological systems and present a novel materials design approach for wearable and implantable biosensors of the future [61]. Recently, there have been significant advances in the use of different materials to prepare the hydrogel-based sensors [53,62], including natural polymers such as cellulose [63], alginate [64], and chitosan [65,66] as well as synthetic polymers such as polyacrylamide [67,68,69] and polyethylene glycol [70]. Furthermore, the incorporation of nanomaterials [71] such as graphene [72], metal nanoparticles [73,74], and MXenes [75] has significantly enhanced the electrical, mechanical, and sensing performance of hydrogels [71,76]. Advances in stimuli-responsive designs have enabled the creation of smart hydrogels capable of responding to multiple environmental triggers [77,78,79,80]. In addition, integration with flexible and wearable electronic systems has expanded the practical applications of these sensors [81,82]. However, when it comes to signal quality, long-term stability, and integrating sophisticated features, conventional hydrogel sensors still have limitations, and the tensile strength (0.028 ± 0.01 MPa) of traditional hydrogels can be enhanced to 14.95 ± 2.69 MPa with a conductivity of (0.2–4 S/m), whereas the incorporation of liquid metals can enhance it to 19,011 S/m, yet such enhancements introduce dispersibility, solubility, and biocompatibility issues [38].

Despite rapid progress in the field, existing reviews have addressed hydrogel-based sensors from narrow or fragmented perspectives. Reviews focusing on individual polymer classes [83,84] cover material properties without linking polymer chemistry to transduction mechanism selection or application performance. Reviews centered on single transduction mechanisms, such as conductive hydrogels in flexible strain sensing [76], omit chemical and optical sensing modalities entirely. Reviews limited to specific application domains such as wearable electrochemical biosensing do not address mechanical, thermogalvanic, or colorimetric platforms [85]. Critically, none of these reviews cover the complete pipeline from polymer composition through fabrication strategy to sensing mechanism and multi-domain application within a single critically evaluated framework. Furthermore, emerging platforms including self-powered thermogalvanic sensors [86], SERS-integrated hydrogel biosensors [87], and MXene/MOF composite electrochemical systems still remain largely unaddressed in the existing literature. The rapid convergence of multimodal, interface-engineered [88], and AI-assisted hydrogel sensor platforms therefore creates an urgent need for a comprehensive and critically integrated review that bridges these gaps. This review fills the aforementioned research gaps via a mechanism-focused, critically appraised overview of hydrogel-based sensors, differing from prior literature in three core aspects. First, it systematically correlates polymeric composition and crosslinking chemistry with the choice of transduction mechanisms and resultant sensing performance. Second, it comprehensively summarizes cutting-edge sensing configurations including self-powered thermogalvanic devices, SERS-enabled sensors [89], and MXene/MOF composite platforms that are rarely covered in previous reviews, incorporating up-to-date publications to outline recent progress in multimodal and interface-engineered wearable biosensors. Third, this work represents the first critical review to integrate polymer design, fabrication protocols, sensing principles, and cross-disciplinary applications spanning biomedical diagnostics and environmental monitoring into a unified analytical framework.

## 2. An Overview of Polymers Used in Hydrogel-Based Sensors

Polymers are crosslinked chemically or physically to form a 3-D network with gel-like properties; this is called a hydrogel. Hydrogels have diverse properties that can be utilized for various purposes, such as absorption and sensing. In short, different polymers crosslinked to synthesize a hydrogel-based sensor with distinctive properties. Also, the polymers adopted for hydrogel fabrication possess unique properties. However, their functional groups can be modified during the crosslinking reaction, thereby achieving substantial improvements in the mechanical, physical, and chemical performances of hydrogels. Moreover, in hydrogel-based sensor networks, these polymers, after crosslinking, have unique sensing capabilities like responsiveness to external stimuli, conductivity, chemical detection, and pH detection, etc., in the hydrogel. Polymers used in hydrogel-based sensors are categorized into natural polymers and synthetic polymers.

### 2.1. Natural Polymers

Cellulose, chitosan, protein, starch, alginate, and their derivatives are the natural polymers used in hydrogel-based sensors [90], and while their hydroxyl-rich molecular architecture confers inherent biocompatibility and biodegradability, it simultaneously limits conductivity, mechanical robustness, and resistance to enzymatic degradation, persistent challenges that constrain long-term stability, selectivity, and scalable manufacturing across all natural polymer classes [90,91]. These polymers are abundantly available in natural resources, making them cost-effective and sustainable options for material development [91]. Among the natural polymers, cellulose is one of the most abundant natural renewable polymers widely utilized in the hydrogel, food, paper, packaging, textile, and biomedical industries [92]. Moreover, its derivatives, such as nanocellulose, cellulose nanofibrils (CNF), and cellulose nanocrystals (CNC), are used for the mechanical reinforcement of hydrogels and in hydrogel-based sensors [93], as both CNF and CNC are made up of semi-crystalline, β-linked D-glucose units that have remarkable mechanical qualities as well as surface chemistry and reactivity that may be altered chemically [93]. Cellulose is inherently hydrophilic because of its abundant hydroxyl groups, while its strong interchain interactions, including extensive hydrogen bonding, contribute to its resistance to dissolution in water and many common solvents [94]. The mechanical strength and the improvements through functionalization or crosslinking have potential for cellulose-based strain sensors [95]. The incorporation of cellulose nanomaterials like CNF with PVA in hydrogel-based sensors results in stretchable, self-healing, and transparent sensors [96].

Bacterial cellulose and plant cellulose used in hydrogel-based sensors are chemically similar, but plant cellulose is associated with the hemicellulose, pectin, lignin, and other biogenic products, whereas bacterial cellulose is a source of pure cellulose. Thus, it requires minimal chemical and mechanical treatment in the preparation of the hydrogel-based sensor, as it can simply be purified through sodium hydroxide treatment. Biosensors made from bacterial cellulose (BC) are used in human computer interface (HCI) applications as well as in wearables due to their ability to integrate seamlessly into textile substrates, owing to their ability to precisely sense various activities performed by people, ranging from delicate physiological signals such as vocalization or breathing, and pulse wave up to larger activities. The creation of intricate HCI systems relies heavily on this characteristic, making them sensitive and easy to use [97]. Furthermore, by incorporating MXene into bacterial cellulose, it is possible to create a hydrogel, where both of them would enhance electrical conductivity and mechanical robustness. This hydrogel is highly responsive in terms of sensing sensitivity and signal stability. Thus, hydrogels can be utilized in suitable wearable flexible sensors for detecting motions and vocalization [98]. Carboxymethyl cellulose (CMC) is another natural polymer that is applied to hydrogels to detect motions owing to its characteristics in being able to thicken, disperse, or stabilize formulations [99,100]. Xie et al. (2025) discussed that CMC-based hydrogel sensors are striking because carboxymethyl cellulose offers low cost, biocompatibility, hydrophilicity, and versatile network formation, enabling soft and deformable sensing platforms; however, their broader practical use is often limited by weak intrinsic conductivity, moisture sensitivity, and the need for additional functional components to achieve high sensitivity and long-term stability [101].

Alginate is also widely used in hydrogel-based sensors due to its biocompatible, hydrophilic properties and easy gelling by multivalent ions such as Ca^2+^. Thus, alginate-based hydrogel is preferred when the device needs a high water content and permeability to ions or analytes [102,103]. Sun et al. (2023) synthesized an alginate-based hydrogel sensor that can be used as a wearable and underwater strain sensor for monitoring human motion and communication [104]. Alginate in the hydrogel can provide abundant carboxyl and hydroxyl groups that participate in hydrogen bonding with PAM. Moreover, due to the alginate, the hydrogel exhibits anti-swelling performance. Thus, the sensor can be used for underwater motion monitoring, including breathing, knee bending, elbow bending, and pulse detection during swimming. Another outstanding characteristic of alginate is its capacity to form hydrogen bonds that may increase the physical and mechanical strength of the hydrogel, including elasticity and toughness [105]. These properties come in handy, especially in designing strain sensors to be used in wearable devices [106]. Despite their excellent biocompatibility and the structural mimicry of the extracellular matrix, alginate-based hydrogels suffer from critical limitations for biomedical and sensing applications [102]. These include low mechanical strength leading to adhesion failure, poor thermal stability, and uncontrolled degradation causing inconsistent sensor performance [103] and a lack of intrinsic conductivity or stimuli-responsive sensitivity [104] that necessitates conductive fillers (e.g., graphene oxide, MXene), increasing fabrication complexity and potential toxicity [102,103]. Additional challenges include poor cell adhesion due to the absence of cell-recognition motifs [102], excessive swelling [103,104], batch-to-batch variability, and sensitivity to sterilization methods [102]. These limitations highlight the need for hybrid systems and smart functionalization strategies to realize the potential of alginate-based materials [104].

Protein-derived hydrogels have attracted substantial research interest, given that gelatin and collagen possess favorable mechanical robustness and an inherent bioactivity, properties highly desirable for biosensors interfaced with biological systems [107]. For instance, a strain-insensitive and environmentally stable collagen-based multifunctional hydrogel (CDPAP) sensor was developed using collagen, acrylic acid (AA), dialdehyde carboxymethyl cellulose (DCMC), 1,3-propanediol, and AlCl_3_. The resulting hydrogels demonstrate exceptional stretchability, repeatable adhesion, self-healing capabilities, freeze resistance, and biocompatibility. However, protein-based hydrogel sensors, particularly those based on collagen, face several inherent challenges, including low water solubility, susceptibility to denaturation in acidic, alkaline, or high-temperature environments, and a lack of intrinsic self-healing and environmental adaptability [108]. According to Liu et al. (2024) natural polymers offer advantages of biocompatibility, biodegradability, and low cost, but their performance in hydrogel-based sensors is often limited by low conductivity, poor mechanical strength, and insufficient environmental stability compared to synthetic polymers [109,110]. However, to achieve specific traits such as excellent mechanical properties, high electrical conductivity, and strong adhesion, natural polymer materials are blended with synthetic materials. However, this approach tends to reduce the biodegradability and biocompatibility of natural hydrogels [109].

Table 1 demonstrates the broad applicability of natural polymer-based hydrogel sensors, a critical examination reveals that no single natural polymer in isolation satisfies the full spectrum of performance requirements demanded by practical wearable applications. A recurring pattern across all five polymer classes is the trade-off between individual functional gains and unresolved mechanical or operational deficiencies. Anti-freezing cellulose formulations extend operational windows to −54 °C [111] and −45 °C [112], yet cycling durability collapses to fewer than 50 and 10 cycles respectively, rendering them impractical for continuous use. Alginate systems achieve the highest strain capacity in the table at 1500% [113] and demonstrate switchable multimodal functionality [114], but their sensitivity to ionic concentration imbalance and the absence of intrinsic conductivity necessitate synthetic conductive fillers, increasing fabrication complexity. Gelatin-based entries reach the highest compressive strength of 1310 kPa [115] and the most capable TENG output of 232 V [116], yet self-healing recovery stalls at 36.7% tensile restoration [115] and water retention drops to ~50% after 13 h at body temperature [116]. Chitosan delivers the strongest adhesion of 327 kPa and broadest signal acquisition [117], but two of its three table entries [118,119] report no self-healing capability. Starch yields the highest GF of 5.93 [120] and the strongest biodegradability of over 85% in 40 days [121], but both entries are entirely additive-dependent for conductivity and mechanical competence. These convergent limitations across structurally distinct natural polymers indicate that the constraints are not incidental but are inherent to the molecular architecture of each class. The most consistently high-performing entries in Table 1, SA/PAM/Gelatin [113], Gelatin/DATNFC [115], BC/CMC/Chitosan [122], and Fish Gelatin/CNF [116] are precisely those employing hybrid natural polymer networks, where complementary chemistries offset individual weaknesses while preserving biocompatibility and sustainability. This points clearly to interpenetrating hybrid network design, rather than single-polymer optimization, as the necessary direction for achieving the simultaneous sensitivity, durability, self-healing, and environmental stability required of clinically viable natural polymer-based flexible biosensors. These findings collectively demonstrate that the molecular architecture of each natural polymer classes the degree of hydroxyl group density in cellulose, the carboxyl group availability in alginate, and the amine reactivity in chitosan, and the amide bond flexibility in gelatin directly governs the crosslinking density, swelling behavior, and conductivity of the resulting hydrogel network, which in turn determines the sensitivity ceiling, response time, and operational stability of the sensor [90,109].

### 2.2. Synthetic Polymers

Synthetic polymer-based hydrogels offer the superior tunability of mechanical properties, network architecture, and chemical functionalization compared to their natural counterparts [128]. Despite their tunability advantages, synthetic polymer-based hydrogels face unresolved scalability constraints including narrow UV curing windows, precise initiator concentration requirements, and multi-step functionalization processes that complicate batch-to-batch reproducibility and limit translation from laboratory to manufacturing scale [128,129]. The most extensively studied synthetic polymer systems for sensing applications include polyacrylamide (PAAm) [130,131], poly(N-isopropylacrylamide) (PNIPAM) [132,133], polyvinyl alcohol (PVA) [43], poly(acrylic acid) (PAA) and poly(ethylene glycol) (PEG) [134]. Synthetic polymers are widely used in hydrogel-based sensors due to their properties like flexibility, wearability, and tunability [135]. Synthetic polymer-based hydrogels have become a key platform for flexible and wearable sensors because they have mechanical properties, biocompatibility, and straightforward functionalization with conductive or responsive components [136]. The characteristics of synthetic polymer-based hydrogels make them suitable for various applications, such as environmental monitoring and healthcare diagnostics [39]. Yu et al. (2023) synthesized a hydrogel where PAA serves as the primary synthetic polymer in this hydrogel system, providing mechanical strength, strain-stiffening behavior, self-healing ability, and low hysteresis. Its carboxyl groups enable dynamic hydrogen bonding with both itself (weak bonds for energy dissipation) and with Stereocomplex poly(ethylene glycol)-b-poly (lactic acid) (sc–PEG-PLA) micelles (strong bonds for strain-stiffening). The resulting hydrogel combined skin-like mechanical properties with high sensing performance, making it suitable for wearable strain sensors [137]. Similarly, Wang et al. (2026) employed PAA as the primary polymer matrix in a PAA/EG/TA@MWCNT hydrogel, where the PAA network enables the uniform dispersion of conductive fillers (TA@MWCNT) and provides mechanical robustness (tensile strength of 1.013 MPa) while the EG imparts an anti-freezing capability (conductivity of 1.47 S/m at −24 °C). The PAA matrix also contributes to strong adhesion (146 kPa), self-healing (84% efficiency), and high sensitivity (GF = 5.865). These studies demonstrate that PAA, through its carboxyl groups and network-forming ability, serves as a versatile synthetic polymer for hydrogel sensors, enabling either strain-stiffening or a balance of conductivity, anti-freeze, and high sensitivity [138]. Acrylamide is another synthetic polymer used in hydrogel-based sensors. He et al. (2025) synthesized a hydrogel-based sensor for wearable motion monitoring and handwriting recognition where AM (Acrylamide) serves as the primary polymer network, providing mechanical strength, toughness, and hydrogen bonding with wood fibers. AMPS (2-Acrylamido-2-methyl-1-propanesulfonic acid) acts as the conductive filler, dissociating to provide ionic conductivity up to 3.18 S/m. The combination of AM and AMPS, reinforced with a delignified wood skeleton, yields a hydrogel with high tensile strength (1.3 MPa), good fatigue resistance (100 cycles), and suitable sensing performance (GF = 0.77) for wearable strain sensors and triboelectric nanogenerators [139]. Moreover, the integration of conductive materials into these hydrogels can further enhance their sensing capabilities, leading to improved performance in real-time monitoring applications. These advancements position synthetic polymer-based hydrogels as crucial components in the development of smart sensors that can adapt to changing environmental conditions and user needs and drive innovation in sensor technology, offering promising solutions for both medical diagnostics and environmental applications [53].

Table 2 summarizes the key properties, and limitations of synthetic polymer-based hydrogels used in strain and wearable sensing applications. Among the polymers surveyed, poly(HEAA-co-SBAA) exhibits the highest stretchability (4000–5000%), while PNIPAM offers exceptional solvent stability (>400 days) and fast response (64 ms). PAA demonstrates a unique strain-stiffening behavior (up to ~10.5 MPa) with low hysteresis (η ≈ 0.2), and pAAm/carrageenan enables multi-stimuli sensing (strain, temperature, humidity, gas) with a high gauge factor of 6. PVA, AMPS, and P(SBMA-co-AAm) provides balanced performance with good stretchability (1000–1353%) and self-healing capabilities (3 s to 30 min). These polymers collectively offer a wide range of mechanical, electrical, and functional properties suitable for diverse sensor applications. Despite their advantages, synthetic polymer-based hydrogels face several persistent challenges. Conductivity remains a major limitation, with most polymers requiring conductive fillers (e.g., PEDOT: PSS, LiCl, graphene oxide) that introduce fabrication complexity and potential toxicity. Self-healing ability degrades after multiple cycles (e.g., PVA after 2 cycles), and hysteresis increases at high strains (e.g., PAA > 200%). Environmental stability is another concern for synthetic polymers for instance PNIPAM swells in water and acids, while pAAm/carrageenan requires organohydrogel formation to achieve extreme freezing and drying tolerance. Moreover, fabrication constraints such as UV curing, precise concentration control, and one-pot synthesis at elevated temperatures (60 °C) limits scalability. Additionally, the gauge factor of fully polymeric hydrogels (typically 1.5–4.9) remains lower than that of nanocomposite-based sensors, which can achieve GF values exceeding 100.

Building on these limitations, a critical comparison reveals that no single synthetic polymer excels across all performance categories, and material selection must therefore be guided by application-specific priorities. PAA emerges as the most versatile matrix, as demonstrated by two contrasting examples exploiting dynamic hydrogen bonding with sc-PEG-PLA micelles to achieve 98.5% self-healing efficiency and state-independent sensing with GF = 1.66, prioritizing sensing reliability [137], while tuning the same backbone toward environmental resilience, achieving a tensile strength of 1.013 MPa, an anti-freezing conductivity of 1.47 S/m at −24 °C, and GF = 5.865. This contrast illustrates that network architecture and composite integration are as decisive as polymer identity in determining sensor performance [138]. Acrylamide-based systems demonstrate that similar versatility reinforced an AM/AMPS network with a wood skeleton to achieve a tensile strength of 1.3 MPa, a conductivity of 3.18 S/m, and GF = 0.77, showing that structural reinforcement can partially decouple the mechanical–electrical trade-off inherent to soft hydrogel systems [139].

The stretchability–sensitivity trade-off emerges as the most persistent challenge across all synthetic systems. Poly(HEAA-co-SBAA) achieves the highest stretchability of 4000–5000% but only moderate GF of ~2.0, while systems with moderate stretchability pAAm/carrageenan (950%) [145] and PNIPAM (2512%) achieve higher GF values of 6 and 2.14–4.96 respectively. This inverse relationship reflects a fundamental structural constraint: networks optimized for extreme deformation inevitably dilute conductive pathway density, reducing gauge factor [142]. Collectively, these findings confirm that carboxyl group density, network topology, and filler dispersion homogeneity, rather than polymer identity alone, are the primary molecular determinants of gauge factor, hysteresis, and environmental stability in synthetic hydrogel-based sensors [137,142,145], and that future progress will therefore depend not on discovering a single superior polymer but on developing composite and hybrid architectures that intelligently combine the complementary strengths of multiple synthetic systems [53]. Realizing such architectures, however, requires deliberate control over crosslinking strategy, processing conditions, and device integration aspects governed by fabrication rather than polymer chemistry alone, as examined in the following section.

## 3. Fabrication Strategies for Hydrogel-Based Sensors

The sensing performance, durability, and device integration of hydrogel-based sensors are critically dependent on the structural design and crosslinking strategies employed to form the three-dimensional network. This section outlines the complete manufacturing workflow, and the discussion evaluates the crosslinking approaches, namely chemical crosslinking, physical crosslinking, and composite hydrogel fabrication.

### 3.1. Fabrication Overview

The transition from raw chemical precursors to a fully integrated, functional hydrogel sensor follows a comprehensive, multi-stage translation pipeline. This sequence is designed to control the material architecture from the molecular level up to its macroscale integration into electronic systems.

**Figure 2:**
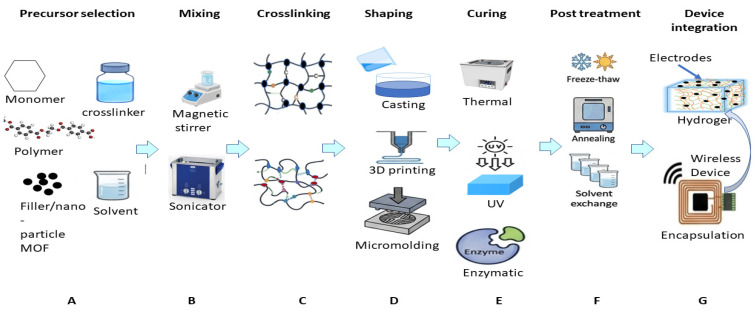
Schematic illustration of hydrogel-based sensor fabrication; (**A**) precursor selection (**B**), mixing (**C**), crosslinking (**D**), shaping (**E**), curing (**F**), post-treatment (**G**), and device integration.

The transformation of raw chemical building blocks into sophisticated, electronically active hydrogel sensors relies on a highly coordinated fabrication pipeline rather than an isolated synthesis event [39,146]. This holistic manufacturing workflow is structured as multiple steps that bridge molecular level polymer chemistry with macro-scale device engineering, ensuring that each step directly modulates the physical and electrical properties of the final sensing platform (Figure 2). The process begins with precursor selection (Stage A), where polymers, monomers, crosslinkers, functional fillers, and solvents are systematically selected to establish baseline parameters such as mechanical compliance, biocompatibility, and stimuli-responsiveness [78,147]. To prevent the performance-limiting nanomaterial agglomeration typical of advanced composite networks, these precursors undergo intensive mixing (Stage B) via mechanical stirring or high-energy sonication to achieve a uniform, liquid pre-gel solution [75,148]. Crucially, because high-resolution sensor architectures and intimate electronic interfaces are required, shaping (Stage D) is executed while the material is still in its fluid state. The liquid pre-gel is patterned into its required geometric form using techniques such as mold casting directly onto electrode arrays, 3D printing extrusion, or high-precision micro-molding [149,150,151,152,153]. However, in casting and micromolding, the liquid precursor solution is shaped before crosslinking is triggered [149]. Once the desired shape and substrate wetting are achieved, Crosslinking (Stage C) and Curing (Stage E) are triggered simultaneously or sequentially via external stimuli (such as thermal energy, ultraviolet radiation, or enzymatic activation). This locks the mobile polymer chains into a cohesive, non-flowing three-dimensional matrix, permanently trapping the conductive components within the defined geometry [154,155,156,157]. To refine the structural integrity and environmental stability of the freshly solidified network, a post-treatment (Stage F) phase incorporating freeze–thaw-induced crystallization, thermal annealing, or solvent exchange to leach out cytotoxic, unreacted monomers is deployed [158,159,160,161]. Finally, the standalone hydrogel is subjected to device integration (Stage G), where it is structurally combined with hardware elements like flexible electrodes, sealed in an encapsulation barrier to prevent dehydration [162], and linked to wireless communication modules to achieve real-time, untethered signal transduction [163,164].

### 3.2. Chemical Crosslinking Methods

Chemical crosslinking involves the formation of irreversible covalent bonds between polymer chains, establishing a permanent three-dimensional network [39,146]. This fabrication approach provides unparalleled dimensional reliability and resistance to hydrolytic degradation [147]. However, the inherent network heterogeneities introduced during covalent gelation fundamentally constrain the linear dynamic range and signal recovery kinetics of the resulting sensor. Unlike physical crosslinking, which relies on hydrogen bonding or electrostatic attraction, chemical crosslinking forms permanent bonds, resulting in better shape retention and resistance to dissolution. Jiang et al. (2023) discussed the covalent incorporation of acryloyl-terminated hyperbranched PCL into the polymer network during copolymerization, thereby establishing a stable framework that enhances hydrogel robustness and supports the synergistic action of dynamic non-covalent interactions [165]. Similarly, Du et al. (2021) discussed that chemically crosslinked hydrogels are important structural platforms for optical ion sensing because irreversible covalent networks provide stable three-dimensional matrices with sufficient mechanical integrity, water retention, and capacity for immobilizing receptors and signal transducers. At the same time, chemically crosslinked systems often require multistep functionalization, may exhibit batch-to-batch variability, and are frequently fabricated by UV-induced polymerization, which can photodamage entrapped sensing components. Moreover, strongly crosslinked hydrogels can restrict analyte diffusion, leading to slow response kinetics and prolonged assay times [50]. Common directions of chemical crosslinking include free radical polymerization [154,166,167], Schiff base reactions [168,169], enzymatic crosslinking [157], and click-chemistry approaches such as Diels–Alder coupling [170,171]. Free radical crosslinking is a polymerization method in which initiators (such as ammonium persulfate, APS) decompose under thermal, UV, or redox activation to generate highly reactive free radicals. These radicals attack vinyl monomers (e.g., acrylamide) and crosslinkers (e.g., N, N′-methylenebisacrylamide, MBAA), propagating chain reactions that form covalent bonds between polymer chains, ultimately creating a three-dimensional hydrogel network. However, free radical crosslinking in the work of Song et al. (2026) exhibits residual toxicity, no self-healing, non-biodegradability, narrow processing windows, and fabrication constraint [129]. Moreover, in more advanced sensor designs, dynamic covalent bonds such as imine, boronic ester, hydrazone, and disulfide linkages are also used to introduce self-healing and adaptability [172].

Enzymatic crosslinking provides a mild route to hydrogel formation by connecting polymer chains. Polymers containing tyrosine, tyramine, dopamine, or aminophenol groups gel rapidly upon H_2_O_2_/HRP oxidation, through radical interactions at the ortho-positions of phenolic hydroxyl groups [173]. Using this method, recently Hasturk et al. (2020) successfully modified regenerated silk with tyramine-substituted gelatin or silk, creating hydrogels ideal for mammalian cell culture and encapsulation [174]. Also, Wang et al. (2022) demonstrate that the enzymatic crosslinking of unmodified silk fibroin using HRP/H_2_O_2_ in a weakly acidic buffer (HAc/NaAc, pH 6.0) produces a high-performance, biodegradable, and biocompatible hydrogel suitable for electronic skin applications [175]. This approach eliminates the need for the tyramine chemical modification required in the Hasturk et al. (2020) study, simplifying the fabrication process while achieving excellent mechanical and sensing properties [174]. Another study by Tordi et al. (2026) revealed that microbial transglutaminase (TG), a food-grade enzyme produced by microbial fermentation, provides an effective and environmentally benign route to covalently stabilize gelatin networks. TG catalyzes the formation of ε-(γ-glutamyl)lysine isopeptide bonds between the side chains of glutamine and lysine residues on gelatin, establishing a permanent, enzyme-crosslinked network [156]. However, Enzymatic crosslinking offers mild conditions, biocompatibility, and sustainability, yet HRP-based systems require H_2_O_2_ and suffer from slow gelation, chemical modification needs [174], or synthetic polymer blending [175]. While TG-based crosslinking overcomes these limitations, it still exhibits lower sensitivity (GF 2.86 vs. >10) and a limited linear sensing range (300% strain) [156].

Schiff base crosslinking is a chemical crosslinking method in which a dialdehyde crosslinker (glutaraldehyde) reacts with free amine groups (-NH_2_) on polymer chains (gelatin) to form covalent imine bonds (C=N), creating a permanent three-dimensional hydrogel network. A covalent crosslinking mechanism, wherein glutaraldehyde’s aldehyde groups react with gelatin’s amine groups to form imine bonds, produces a permanently crosslinked, mechanically durable, and deformable hydrogel network suitable for multifunctional robotic skin applications. Crosslinking enables the scalable fabrication (52 × 23 cm), high deformability (100% stretching), fatigue resistance (500 cycles), low compressive stress (1.5–2.4 kPa), and flame retardancy (>60 s at 600–850 °C) of Multi-Walled Carbon Nanotubes (MWNT)-gelatin hydrogels, but suffers from a narrow processing window (6–9 min), dehydration-induced performance loss (GF drops from 0.99 to 0.22), MWNT aggregation at high loading (7 wt.%), and non-linear compressive sensing [176]. In hydrogel sensors, chemical crosslinking is important because it generally improves mechanical stability, network integrity, and long-term sensing reliability [38,177,178]. This is especially useful in biosensors and wearable devices, where the hydrogel must remain attached to the substrate and preserve its structure during swelling, deformation, or repeated use. At the same time, the crosslinker type and concentration strongly influence the hydrogel’s morphology, stability, and sensor performance [36,164]. Chemical crosslinking forms permanent covalent bonds between polymer chains, providing dimensional reliability, resistance to hydrolytic degradation, and long-term structural integrity which are essential for wearable and implantable sensing applications [39,146]. Common strategies include free-radical polymerization, Schiff base reactions, enzymatic crosslinking, photo-crosslinking, and click chemistry, while dynamic covalent bonds such as imine, boronate ester, and disulfide linkages introduce self-healing and stimuli-adaptability [172]. Table 3 summarizes these methods by bond type, reversibility, gelation conditions, and key advantages and limitations.

Chemical crosslinking strategies span covalent and dynamic networks, each governing sensor performance differently. Free radical polymerization, thiol-ene click, carbodiimide coupling [113,176], photo-crosslinking, and enzymatic crosslinking deliver superior mechanical stability but sacrifice self-healing. Dynamic covalent bonds in Schiff base and boronic ester enable self-healing critical for wearable sensors under cyclic loading, but remain pH-sensitive, limiting reliability in physiologically variable environments. Among permanent methods, free radical polymerization is most established but exhibits residual toxicity, non-biodegradability, and narrow processing windows. Photo-crosslinking offers spatial control and rapid curing, yet is restricted by light penetration depth. Carbodiimide coupling achieves strong bioconjugation but requires a multistep process. Enzymatic crosslinking under physiological conditions offers the strongest biological safety (Table 3), with TG-crosslinked gelatin providing a sustainable fabrication route, though yielding only GF 2.86, well below nanocomposite sensors where GF exceeds 10 [156]. Among dynamic methods, Schiff base crosslinking enables large-scale fabrication up to 52 × 23 cm with 500-cycle fatigue resistance, but suffers a narrow processing window of 6–9 min and dehydration-induced GF loss from 0.99 to 0.22 [176]. Boronic ester crosslinking enables fast reversible self-healing under alkaline conditions but remains unstable under acidic physiological environments [181]. Critically, no single chemical crosslinking strategy simultaneously satisfies mechanical robustness, self-healing, fast response, and biological safety. Fabrication constrains UV curing, multistep functionalization, and narrow gelation windows, collectively limiting manufacturing consistency and reproducibility. Strongly crosslinked networks additionally restrict analyte diffusion, prolonging response times in electrochemical and optical sensors [50]. While hybrid chemical approaches combining permanent and dynamic bonds are increasingly adopted, they cannot fully replicate the dynamic reversibility, injectability, and mild processing conditions achievable through non-covalent interaction capabilities central to physical crosslinking, as examined in the following section.

### 3.3. Physical Crosslinking Methods

Physically crosslinked hydrogels form networks through non-covalent interactions such as freeze–thaw-induced crystallization, ionic coordination, hydrogen bonding, hydrophobic association, and biopolymer self-assembly [53,155,186]. In contrast to permanently covalently crosslinked systems, physically crosslinked hydrogels are generally prepared under mild conditions and often exhibit attractive features, including self-healing capability, recyclability, injectability, and favorable biocompatibility [187]. These characteristics make them particularly appealing for wearable, flexible, and biointegrated sensing platforms [85,164]. However, the dynamic and relatively weak nature of these interactions may also lead to limitations such as creep, stress relaxation, dehydration, and insufficient long-term structural stability under repeated mechanical loading or harsh environmental conditions. Therefore, the choice of physical crosslinking mechanism must be closely aligned with the intended sensing function and operating environment [85]. Freeze–thaw crosslinking is widely used in hydrogel-based sensors. Freeze–thaw physical crosslinking can enhance toughness, elasticity, and network stability while preserving the flexibility required for strain-responsive sensing applications [158]. Sun et al. (2024) reported that freeze–thaw cycling/annealing during synthesis accelerated the hydrogel film, which is a great breakthrough in hydrogel film production and its application in wearables [159]; however, the freeze–thaw method can be time-consuming as it takes more time to cyclically freeze and thaw. Moreover, it shows low transparency, which is not reliable for flexible sensors. It is reported that this method can produce an unstable gel network, leading to an overall material reliability [160]. Ionic coordination is another type of physical crosslinking used in the fabrication of a hydrogel-based sensor. The fabrication methodology has proven to be important due to its silent features like reversible network formation, tunability, self-healing, and wet surface adhesion. However, the performance of such a hydrogel-based sensor is constrained by pH, ion sensitivity, the complexity of ion-specific coordination behavior, and time-dependent mechanical relaxation [188].

Hydrogen bonding between functional groups such as phenolic hydroxyl, carboxyl, and amide groups enables fully reversible network formation with strong self-healing and adhesion properties [189]. Hydrophobic association through the surfactant-mediated micellar aggregation of hydrophobic monomers provides exceptional stretchability and toughness through dynamic chain sliding mechanisms [190,191]. Biopolymer self-assembly via π–π stacking, hydrogen bonding, and ionic interactions offers an additive-free route to porous conductive networks, though at the cost of lower mechanical strength [192]. Table 4 summarizes these five physical crosslinking mechanisms by interaction type, reversibility, key advantages, and main limitations.

Table 4 compares five physical crosslinking mechanisms for hydrogels. All offer advantages over chemical crosslinking, including superior biocompatibility, dynamic reversibility, and the absence of toxic crosslinkers. However, each presents distinct trade-offs. Freeze–thaw crystallization provides excellent cyclic stability (1000 cycles) without additives but is thermally sensitive above 60 °C. Ionic coordination enables rapid gelation and high ionic conductivity (0.53 S/m) for bioelectrodes, yet risks ion leaching in physiological fluids. Hydrogen bonding delivers remarkable self-healing (97%) and adhesion (27.42 kPa) but requires precise crosslinker control and weakens under high strain. Hydrophobic association achieves the highest stretchability (1400%) and toughness (1.44 MJ/m^3^), albeit with surfactants that may limit biocompatibility. Biopolymer self-assembly offers an additive-free route to porous networks with high conductivity, but at the cost of lower mechanical strength. In summary, no single mechanism is universally optimal; the choice must be guided by application priorities, cyclic stability, conductivity, self-healing, stretchability, or additive-free fabrication. Emerging trends favor hybrid systems that combine multiple physical interactions to synergistically achieve multifunctional properties. Critically, Table 4 confirms that no single physical crosslinking mechanism simultaneously satisfies stretchability, conductivity, self-healing, and biocompatibility. Freeze–thaw provides 1000-cycle stability but fails above 60 °C [193], ionic coordination achieves 0.53 S/m conductivity but risks ion leaching [193], hydrogen bonding delivers 97% self-healing but weakens under high strain [189]; hydrophobic association reaches 1400% stretchability but requires biocompatibility-limiting surfactants [191] and biopolymer self-assembly offers an additive-free route at the cost of mechanical strength [192]. Hybrid networks combining multiple non-covalent interactions are therefore increasingly necessary and where even these remain insufficient, composite fabrication strategies that embed functional nanomaterials into the polymer matrix provide a complementary solution, as examined in the following section.

### 3.4. Fabrication of Composite Hydrogel-Based Sensors

Composite hydrogel-based sensors are engineered by embedding conductive or functional nanomaterials into a three-dimensional hydrogel matrix. This architecture successfully combines the structural flexibility of a soft polymer backbone with advanced electrical, magnetic, or catalytic performance. Unlike homogeneous ion-conducting hydrogels, the performance of these composite systems relies on establishing continuous, percolative conductive pathways and maintaining strong, stable chemical interactions across the nanofiller–polymer interface [148]. Recently, there have been significant innovations in the fabrication of the composite hydrogels, such as functional nanophase embedding, as demonstrated by Wang et al. (2023), who studied the synthesis of a highly responsive sensor by embedding gelatin-modified MXene nanosheets within a three-dimensional polyacrylamide (PAAm) network. In this design, the gelatin coating acts as a stabilizing sheath that protects the MXene sheets from environmental oxidation and restacking while simultaneously serving as an interfacial mediator that couples the fillers to the PAAm matrix. The resulting material exhibits high toughness, stretchability, resistance to mechanical hysteresis, and reproducible sensing signals [194]. Similarly, Chen et al. (2024) introduced the heterogenous bilayer assembly, an advanced colorimetric gas sensor, by sequentially assembling a PVA/boric acid/Pb^2+^ analyte-sensing layer directly onto a tough PAM/sodium alginate supporting substrate. This structurally and chemically differentiated bilayer design allows for the independent optimization of gas adsorption and optical transduction, enhancing overall sensing efficiency, structural reusability, and environmental stability [195].

Recent advancements in this domain have explained the criticality of in situ filler reduction and binary-solvent templating to mitigate the pernicious effects of nanomaterial agglomeration and environmental dehydration. For instance, the integration of Pd@CeO_2_-anchored graphitic carbon nitride frameworks within alginate matrices has demonstrated nanomolar detection limits (0.64 nM) for neurochemical analytes via enhanced charge transport [196], while glycerol-assisted cryogelation has preserved gauge factors approaching 9.18 at sub-zero temperatures (−20 °C) by suppressing ice crystallization through strong hydrogen bonding between polyhydroxyl groups and water molecules. It is discussed that the ionic liquid, glycerol, and dynamic interchain interactions are jointly engineered to improve anti-freezing behavior, water retention, mechanical durability, and sensing stability, thereby enhancing hydrogel sensor reliability in harsh environments [161]. Collectively, these fabrication advancements and innovations from gelatin-stabilized MXene embedding and heterogeneous bilayer assembly to in situ filler reduction, binary-solvent templating, and glycerol-assisted cryogelation demonstrate that composite hydrogel fabrication is not a single strategy but a design space in which nanofiller identity, interfacial chemistry, matrix architecture, and environmental conditioning must be co-optimized to achieve target sensing performance. Critically, the conductive pathway density, interfacial coupling strength, and network homogeneity established during composite fabrication directly determine the transduction efficiency, sensitivity ceiling, and operational stability of the resulting sensor properties that cannot be recovered or compensated by downstream device engineering once the composite architecture is fixed [71,75,164]. Taken together, chemical crosslinking, physical crosslinking, and composite nanomaterial integration each govern distinct aspects of sensor performance, structural permanence, dynamic reversibility, and electrical enhancement respectively, and their intelligent combination determines the transduction efficiency, sensitivity, and operational stability of the final device. These material-level decisions directly establish the performance boundaries examined through specific transduction mechanisms in the following section.

## 4. Sensing Mechanism

**Figure 3:**
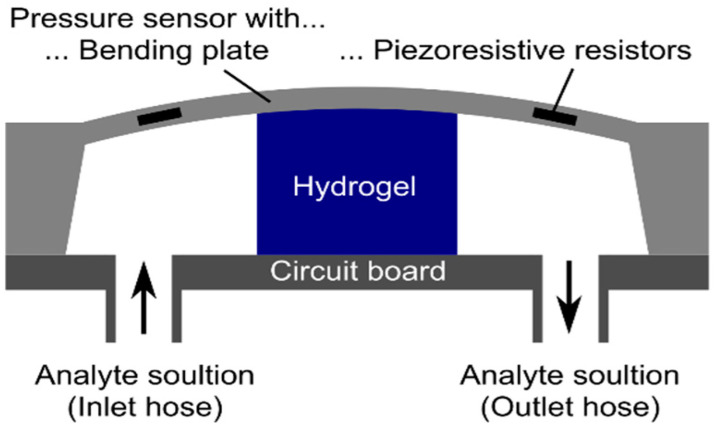
Schematic construction of hydrogel-based piezoresistive pressure sensor [200,201]. Copyrights: image has been taken and reproduced from © 2024, MDPI.

Hydrogel-based sensors work by converting a physical, chemical, and biochemical stimulus into geometrical, biological, electrical, and optical output through a hydrated polymer network [62]. The hydrogel-based sensors can be divided into two main types, based on how the hydrogel functions within the sensor: The active stimuli-responsive hydrogel sensors, which undergo spontaneous volume or phase changes when exposed to physical or chemical stimuli [197], and composite hydrogel sensors, which are made of hydrogel matrices combined with active or responsive materials [198]. The sensing of a hydrogel-based sensor is due to three core features: the network deforms easily under external force, the water-rich phase supports ion transport and analyte diffusion, and the polymer chains can be functionalized with conductive fillers, redox couples, enzymes, aptamers, or optical probes [40]. Recent studies reveal that hydrogel sensing can be classified into physical transduction mechanisms such as piezoresistive, capacitive, piezoelectric, triboelectric, and related pressure-sensing modes. Wang et al. (2024) discussed thermal self-powered sensing, such as thermogalvanic sensing [86] and chemical/biochemical transduction (electrochemical, fluorescence, colorimetric, SERS, and related analyte-responsive modes) [199]. Figure 3 shows a diagram of the hydrogel-based piezoresistive ammonia sensor. A circular hydrogel that responds to ammonia is placed under a piezoresistive pressure sensor chip and attached to the supporting circuit board. Inlet and outlet channels let solutions with analytes in them be exposed in a controlled way. When ammonia comes into contact with the pH-sensitive poly(AAc-co-DMAEMA) hydrogel, it swells because the ammonia makes the surrounding medium more alkaline. The hydrogel is limited by the shape of the device, so when it expands it creates mechanical pressure that pushes the pressure-sensor diaphragm out of the way. The piezoresistive transducer turns this change in shape into a change in output voltage. This lets us indirectly measure the concentration of ammonia through the pressure of the hydrogel swelling. The figure illustrates the principal chemo-mechanical-to-electrical transduction mechanism that forms the foundation of the sensor concept [200,201].

Despite their high sensitivity and simple signal acquisition, piezoresistive hydrogel sensors are subject to several critical limitations that must be considered in practical deployment. Irreversible resistance shifts occur after forced removal due to conductor sliding and viscoelastic stress relaxation within the hydrogel network, compromising baseline stability over extended monitoring periods [149]. Sensitivity non-linearity is a further concern, and pyramid-structured piezoresistive sensors show progressive sensitivity decline from 2.27 kPa^−1^ at low pressure (0–0.5 kPa) to 0.18 kPa^−1^ (0.5–2 kPa) and 0.021 kPa^−1^ (2–5 kPa) due to contact area saturation at higher pressures, requiring precise microstructure fabrication that is difficult to replicate at scale [151]. For strain sensing configurations, mild signal drift arising from the viscoelastic properties of the encapsulation layer accumulates under cyclic loading, and chemically crosslinked variants exhibit permanent structural damage beyond their elastic limit, making physically crosslinked systems preferable despite their comparatively lower sensitivity. Ionic piezoresistive hydrogels additionally show lower conductivity than electronically conductive hydrogels, and the capacitive mode of dual-mode systems becomes entirely unresponsive above 100% strain [150]. Across all piezoresistive sub-types, water evaporation requires encapsulation strategies that add fabrication complexity and may restrict the mechanical freedom required for conformal skin-contact applications. Addressing these limitations requires humidity controlled encapsulation, temperature compensation elements, and dual-mode sensing architectures capable of decoupling mechanical and environmental contributions to the resistance output signal [149,150,151].

The most common mechanism is piezoionic sensing. In this mode, stretching, compression, or bending changes the internal conductive pathways of the hydrogel [202]. In electronically conductive hydrogels, deformation changes the spacing of conductive fillers or percolation channels, altering resistance [203]. In ionic hydrogels, deformation changes ion migration distance, ion distribution, and local polarization, which also changes the measured resistance or current [202]. This is why hydrogel strain and pressure sensors are usually very sensitive to subtle body motion. Recent reviews also emphasize that ionic conductive hydrogels are especially valuable for skin-interfaced sensors because their conduction resembles biological ion transport more closely than conventional rigid electronic materials [40,202]. Capacitive sensing is another type of sensing, where a hydrogel-based sensor exhibits a response to sense the stimuli responses [204,205], detecting a change in capacitance due to a change in external stimuli [206]. Currently, such sensors are parallel plate capacitors with a dielectric layer situated between them. The mathematical equation for the capacitive sensor is as follows:

(1) $$C=\frac{\varepsilon_{r}\varepsilon_{0}A}{d}.$$

where C is the real-time capacitance, **ε**_0_ is the vacuum permittivity (8.854 × 10^−12^ F/m), **ε_r_** is the relative dielectric constant of the dielectric layer, A is the effective area of the two conducting electrode plates, and d, is the distance between the two electrodes. The equation indicates that C is affected by **ε_r,_** A, and d. External stimuli (such as applied pressure) can alter any of these parameters, resulting in a measurable change in capacitance. Specifically, the dielectric constant, **ε_r_**, changes due to deformation or material property variations. Electrode area (A) can vary if the electrodes stretch or compress under stress and plate separation (d) usually decreases under applied pressure, increasing capacitance. Thus, the capacitive pressure sensor converts mechanical stimuli into electrical signals by detecting changes in capacitance caused by variations in these three parameters. The variable influencing the sensitivity in such sensors is the relative change in capacitance that occurs under a specified force. The sensitivity can be enhanced by maximizing the relative change in capacitance, keeping the applied forces in a constant range [203]. Chang et al. (2021) discussed how in flexible pressure sensors capacitive sensing exhibits low sensitivity and low anti-interference capability, although it produces low noise and can respond to static forces [207].

**Figure 4:**
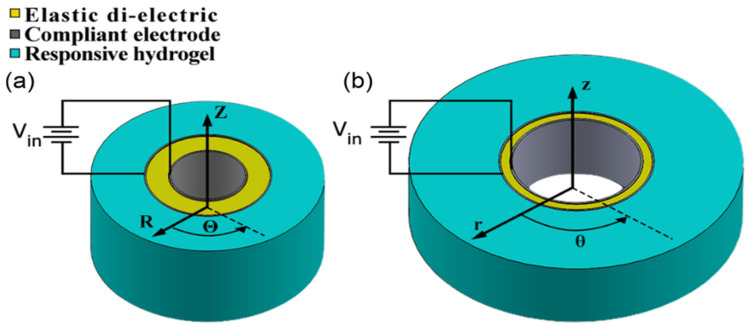
Hydrogel-based capacitive sensor in initial state (**a**) and (**b**) when exposed to ammonium concentration [208]. Copyrights: image has been reproduced under the Creative Commons CC-BY-NC-ND license by Wiley.

Figure 4 shows how the hydrogel-based capacitive ammonium sensor works in theory. Without ammonium, the hydrogel-dielectric assembly stays at its equilibrium shape and has a baseline capacitance. When ammonium is added, ion-induced dissociation in the ionic hydrogel raises the fixed-charge density and creates osmotic pressure, which makes the hydrogel swell. The swelling causes the nearby compliant dielectric layer to deform radially, which brings the electrodes closer together and increases the measured capacitance. So, the figure shows how the proposed device turns changes in the hydrogel’s physicochemical properties caused by ammonium into a measurable capacitive signal [208]. It is evident that capacitive sensing in hydrogel-based sensors offers several advantages; however, significant limitations persist. These include constrained electrode geometry, low baseline capacitance, high output impedance, and parasitic interference [149]. Capacitance plateau above 1 mM for NH_4_^+^ detection requires functional groups with pKa ~8, with experimental validation still needed for real-world conditions [208]. Decreasing sensitivity at higher pressure ranges (dropping to 4.11 MPa^−1^ at 0.6–1 MPa) necessitates surface roughening, graphene integration, and encapsulation to prevent dehydration [209]. These limitations hinder adoption in applications demanding fast, linear, and robust performance and need to be addressed [149,208,209].

**Figure 5:**
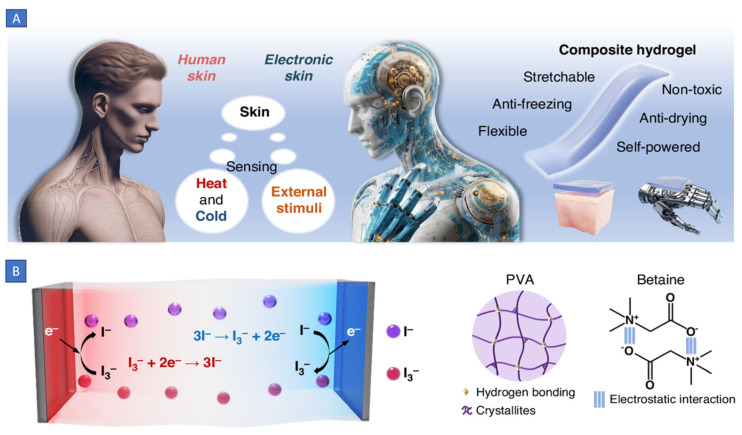
(**A**) Hydrogel acts like skin, perceiving temperature and touch. (**B**) Thermoelectric conversion mechanism and composition of hydrogels [86]. Copyrights: image has been reproduced under the Creative Commons CC-BY-NC-ND license by Springer Nature.

Figure 5A depicts a schematic diagram of the thermogalvanic hydrogel patch resembling skin for the self-powered sensing of temperature and strain. The electronic skin has fundamental sensing abilities, like human skin, such as sensing temperature and tactile stimuli. The hydrogel exhibits a self-sensing mechanism by integrating thermoelectric and piezoresistive effects within a flexible polymer network. As shown in Figure 5B, when a temperature gradient is applied the I^−^/I_3_^−^ redox couple undergoes oxidation and reduction at the two electrodes, generating a continuous electrical output through ion migration and cyclic redox reactions. In addition, external pressure or deformation change the internal conductive pathways of the hydrogel, leading to a measurable variation in electrical resistance for tactile sensing. Owing to its porous PVA-based network with betaine, the hydrogel also provides efficient ion transport, flexibility, and improved water-retention stability, enabling reliable self-powered sensing [86]. Hydrogels are also increasingly used in self-powered sensing, where the material converts mechanical or thermal energy directly into signals. In thermogalvanic hydrogels, a redox couple inside the gel generates an electrical response from a temperature gradient [210].

**Figure 6:**
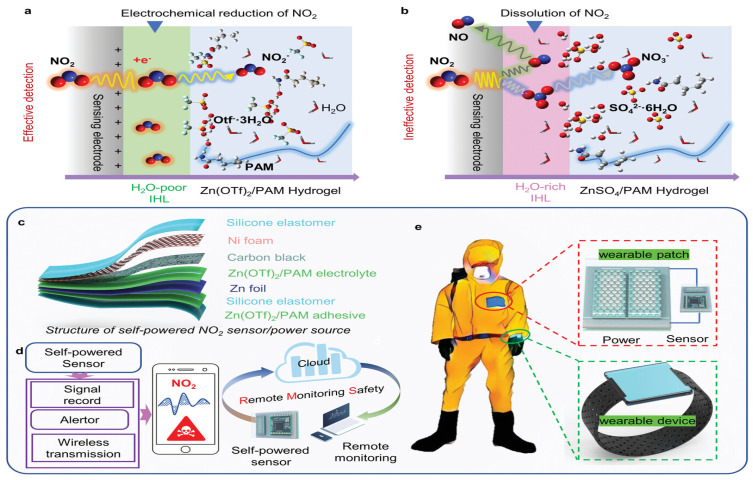
Working principle, structure design, and application of hydrogel-based self-powered NO_2_ sensor: (**a**) effective transfer via hydrophobic interface (H_2_O poor) enabled OtF^−^ ions; (**b**) ineffective charge transfer due to NO_2_ dissolution at H_2_O rich interface; (**c**) sensor structure with perforated silicone top layer; (**d**) wearable self-powered sensor; (**e**) portable wearable device for real-time NO_2_ monitoring [212]. Copyrights: image has been taken and reproduced from © 2023, Wiley.

Chemical sensing mechanism is another type of sensing that exists in the hydrogel-based sensors. The sensing mechanism is usually stimulus-induced swelling/deswelling coupled to ion transport or probe response. When humidity, pH, and gas molecules enter the hydrogel, they change the hydrogen bonding, osmotic pressure, dissociation of functional groups, and water uptake. These changes affect conductivity, impedance, fluorescence, or color, which can be used for sensing. Humidity can be sensed through this sensing mechanism-based hydrogel [147]. Song et al. (2024) discussed how the humidity-activated ammonia sensor showed that the conduction mechanism can shift with relative humidity: proton hopping dominates at low relative humidity (RH), proton conduction occurs through a continuous water film at moderate RH, and ion migration dominates at high RH, while ammonia reacts with water and carboxyl groups to increase ion generation and conductivity [211]. Figure 6a–c schematically summarizes the system-level design and operating principle of the self-powered NO_2_ sensor that uses a hydrogel. Adding amphiphilic OTf^−^ anions make a water-poor inner Helmholtz layer form at the cathode–hydrogel interface. This stops NO_2_ from dissolving and encourages direct interfacial electrochemical reduction, which improves charge-transfer efficiency and gas sensitivity. On the other hand, a water-rich interface makes sensing less effective because dissolved NO_2_ goes through competing interfacial processes. The figure also shows the layered structure of the Zn/hydrogel/carbon device, its flexible shape that is protected, and how it works with a small BLE-based wireless module for real-time NO_2_ monitoring that can be worn. Moreover, interfacial molecular engineering is combined with a self-powered flexible device platform for high-performance gas sensing [212].

In hydrogel-based electrochemical sensors, interaction between the analyte and the hydrogel matrix results in alterations in the hydrogel properties such as swelling, ion transport, and charge transfer, which are then measured as electrical responses. These electrical responses are detected through electrochemical methods such as voltammetry, amperometry, and impedance [213]. In addition, Yun et al. (2025) describe hydrogel microneedle sensors as a key route for electrochemical and optical biomarker monitoring [214]. According to Golshahi et al. (2026) MXene-conductive hydrogels enable electrochemical sensing through dual conduction mechanisms, electronic conductivity via percolated MXene networks, and ionic conductivity through the hydrated polymer matrix, allowing the sensitive detection of metabolites, neurotransmitters, and electrolytes in sweat, urine, and interstitial fluid [215]. Similarly, Yao et al. (2026) highlight that hydrogels enable multimodal bioelectronic systems through conformal skin adhesion (via catechol chemistry), dual ionic/electronic conduction for simultaneous sensing and stimulation, and stimuli-responsive behaviors (to humidity, pH, temperature, and biochemical cues) that dynamically adapt to physiological changes [216]. Studies reveal that compared to pristine hydrogel, MOF and MXenes-based hydrogels are reliable for electrochemical sensing due to better conductivity, electrocatalytic activity, and well-defined active sites. Moreover, MOF-integrated hydrogels have a high surface area, tunable porosity, and high interaction with the targeted molecules [88]. Despite the advantages of electrochemical sensing mechanisms, there are still limitations. Golshahi et al. (2026) report that MXene-specific challenges include oxidation instability, self-stacking that reduces active surface area, and unfavorable –F surface terminations that weaken hydrogel interactions [215]. Similarly, Yao et al. (2026) identify integration challenges including temporal synchronization across modalities, crosstalk, power management complexity, and poor scalability [216]. The main trend in recent years is a shift from single-mode hydrogel sensors toward multimodal, self-powered, and interface engineered platforms that combine mechanical, thermal, and biochemical sensing in one material system [38,210,216]. Table 5 provides a comparative overview of all transduction mechanisms discussed in this section, summarizing their typical sensitivity, response time, and detection limits.

As shown in Table 5, each transduction mechanism occupies a distinct performance niche. Piezoresistive sensing offers fast responses of 70–180 ms with sensitivity up to 2.27 kPa^−1^ as reported by [149,150], but suffers progressive non-linearity at higher pressures. Resistive ionic sensing extends detection to 1585% strain at a limit of 0.1% as demonstrated by Chen et al. (2022) [217], making it suited for large-deformation wearables. Capacitive sensing provides linear pressure responses across 1–1500 kPa [149] but plateaus above 1 mM for chemical analytes [208]. Thermogalvanic sensing is the only self-powered mechanism, achieving thermopower up to 1.44 mV/K [219], though with slower responses of seconds to minutes. Electrochemical sensing delivers the lowest detection limits, with glucose at 0.85 μM and uric acid below 1.2 μM as demonstrated by [220], making it the most analytically precise. Colorimetric sensing responds within 10 s at 0.2 ppm for H_2_S [195], while ratiometric fluorescence achieves 36.6 nM sensitivity for doxycycline [221]. Piezoionic sensing achieves the highest GF of 1242 at 3.12% strain with a 40 ms response [222], the most sensitive self-powered option for subtle mechanical stimuli. It can be concluded that no single transduction mechanism simultaneously optimizes sensitivity, response speed, detection limit, and operational range, a pattern that mirrors the polymer and fabrication trade-offs identified in earlier sections. Each mechanism, however, retains distinct merits that make it uniquely suited to specific sensing demands. The practical realization of these complementary strengths across biomedical and environmental domains is examined in the following section.

## 5. Application of Hydrogel-Based Sensors in Biomedical and Environmental Monitoring

Building upon the broad applications of hydrogel-based sensors, the subsequent sections provide an in-depth examination of specific sensing applications, including pH, temperature, mechanical deformation, and gas detection. These discussions underscore the unique physicochemical properties and recent advancements of hydrogel-based systems, elucidating their potential for precise, real-time monitoring in biomedical and environmental contexts, with a focus on enhancing the functionality and applicability of wearable, flexible sensing platforms.

### 5.1. Biomedical Applications of Hydrogel Based Sensors

#### 5.1.1. pH Sensing

pH-responsive hydrogels generally contain polymer chains with weak acidic or weak basic groups that become protonated or deprotonated depending on the surrounding pH [223]. This alters the electrostatic repulsion among the chains and changes the osmotic pressure within the network, causing structural reorganization and resulting in pH-dependent swelling behavior [224]. Hydrogel-based pH sensors operate via the ionization of weakly acidic or alkaline functional groups incorporated within the polymer matrix [223], primarily the carboxylic acid groups (pKa ~4.5) [225], sulfonic acid groups, and amino groups, leading to the swelling or contraction of the polymer network dependent on the pH level [147,223,224]. The sensors are used in wound treatment and monitoring by sensing the pH which indicates the state of the wound: either healing (acidic pH ~4–6) or infected/chronic (alkaline pH ~7–9) [226]. Kiti et al. (2025) developed a hydrogel-based wound dressing material to indicate bacterial infections in the wound through visual colors. The hydrogel used in the wound dressing can exhibit the colorimetric characteristics of butterfly pea flower extract (BPE). It changes from blue to blue-green at pH 7.4 to 10, and at pH 12.0 it changes from yellow to green; such a feature enables the visual detection of infection status in the wound. Thus, such a hydrogel contains amoxicillin, which can be used as a pH sensor to monitor the wound status and can be prolific for wound dressing applications [227]. Similarly, Albay et al. (2025) synthesized a hydrogel-based sensor for pH detection in wounds through a multilayer wound patch. The methacrylated chitosan (Meth-Chi)/(methacrylated PVA) Meth-PVA hydrogel pH sensor enables continuous wound monitoring with 16.92 mV/pH unit sensitivity across pH 5.15–8.22, four-fold enhanced fluid uptake via an integrated evaporation pad, and stable operation over 15 days (13% deviation), providing early detection of infection-related alkaline shifts (pH > 7.5) for smart, biodegradable wound dressings [228]. The Janus PP/BFD-CP hydrogel dressing enables integrated wound pH monitoring and electrically controlled on-demand therapy for intelligent infection management. The upper CP layer provides real-time pH visualization with a G/B ratio response of Y = −74.85 ln(X) + 162.73 (R^2^ = 0.99) across the physiologically relevant range (pH 5.5–8.5), enabling early infection detection via colorimetric transition from yellow to red. Upon US irradiation (1.5 W cm^−2^, 1 MHz, 5 min), the piezoelectric PP/BFD layer generates a voltage output of 2.83 V and ROS-mediated antibacterial efficacy of 99.7% against *E. coli*, while electrically triggered DHA release reaches 55% within 60 min. Subsequent low-ROS stimulation (1 min US) promotes cell proliferation by 65% and achieves complete wound closure within 10 days in vivo. This diagnostic–therapeutic platform provides a cost-effective, antibiotic-free strategy for smart wound management [229].

Beyond wound monitoring treatment, hydrogel-based pH sensors can be used in wearable physiological monitoring. For real-time physiological monitoring, Odinotski et al. (2022) developed a wearable electrochemical biosensor known as the conductive hydrogel microneedle (HMN) pH meter. This device utilizes dopamine-conjugated hyaluronic acid with PEDOT: PSS to enable on-needle, real-time pH monitoring in interstitial fluid through catechol–quinone redox chemistry via chronoamperometry at 0.4 V. The system achieves 93% in vivo accuracy compared to commercial probes across a clinically relevant pH range of 3.5 to 9. It also demonstrates a mechanical strength exceeding 0.6 N per needle, approximately 250% swelling for efficient ISF uptake, 99% cell viability, and complete skin healing within 10 min. Collectively, these features offer a minimally invasive platform for detecting diabetic ketoacidosis and other pH-related disorders [230]. There have been advancements in the development in applications of hydrogel-based sensors, multifunctional hydrogel-based sensors for instance were developed to address the challenge of developing a single hydrogel sensor with both robust mechanical properties and multifunctional sensing capabilities. Du et al. synthesized a PVA/P(AA-NIPAM)-Fe^3+^ hydrogel which is capable of detecting stress, pressure, temperature, and pH simultaneously while exhibiting a tensile strength of 1.91 MPa and an elongation of 475% [43]. Hydrogel-based pH sensors represent a promising class of soft sensing platforms, capable of translating chemical variations into rapid and reliable optical or electrochemical signals. Their flexibility, biocompatibility, and responsiveness make them highly suitable for applications in wound monitoring, wearable devices, and environmental analysis. Recent progress toward multifunctional sensors further enhances their practical relevance and importance [43,227,229,231]. Addressing these limitations requires the incorporation of dual-network architectures that decouple mechanical robustness from chemical responsiveness, antifouling surface coatings to prevent protein adsorption in biological fluids, and encapsulation strategies that maintain hydration without restricting analyte diffusion [85,230].

#### 5.1.2. Temperature Sensing

**Figure 7:**
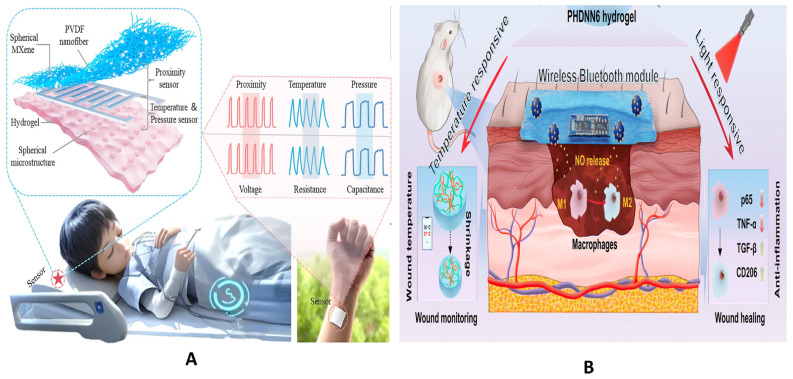
(**A**) MPPT hydrogel application in temperature sensing [234]. (**B**) Temperature and light dual-responsive hydrogel for immunomodulation and wound healing management [235]. Copyrights: (**A**) image has been reproduced under the Creative Commons CC-BY-NC-ND license by Springer Nature; (**B**) image has been taken and reproduced from © 2025, Royal Society of Chemistry.

Temperature is a critical parameter in chemical and physical processes, and its accurate monitoring is essential for maintaining optimal conditions across biomedical, environmental, and industrial applications. Recently, various hydrogel-based temperature sensors have been developed for precise real-time monitoring, offering significant advantages in disease detection, wound management, and continuous health surveillance. To detect the fever, Wang et al. (2021) synthesized a thermoresponsive hydrogel fever detector, which is a wearable colorimetric optical sensor. The sensor has a detection threshold of wrist temperature > 36.2 °C (corresponding to tympanic fever threshold of >37.2 °C). The device provides visual fever alerts at wrist temperature thresholds > 36.2 °C with 20-cycle stability and a raw material cost of <$0.001 per detector. However, key limitations include ambient temperature interference (0.1 °C offset per 5 °C ambient drop), binary rather than quantitative readout, lack of validation on actual fever patients, and exercise-induced false positives. While offering an inexpensive, battery-free solution for continuous mass fever screening during pandemics, clinical validation and environmental compensation strategies remain necessary before widespread deployment [232]. Expanding the hydrogel-based sensors, Yang et al. (2025) developed a wireless hydrogel thermotherapy system with closed-loop temperature control for wound healing. The thermo-responsive NSDH hydrogel enables customizable adhesion (15.58 kPa at 20 °C to 1.93 kPa at 50 °C, >8-fold range) via PNIPAM chain collapse above LCST, allowing robust wound positioning, conformal contact during therapy (42 °C), and benign detachment post-treatment. The anisotropic serpentine mesh heating layer provides uniform heating (ΔT < 1.5 °C across 75% area), 87.73% transparency, and 3.83 Ω resistance, reaching 51 °C at 2 V within 115 s. Integrated wireless electronics enable closed-loop control with ±0.1 °C accuracy. In rat models, the system achieved 99.58% healing at day 14 (15% improvement over control), reducing healing time by 3–4 days. Limitations include slow heating (115 s), opacity at therapy temperature (transmittance drop to 36.5%), a 50 °C detachment requirement, complex synthesis, a lack of human data, and no infection monitoring [233]. Figure 7A illustrates the structure and working mechanism of the multimodal sensing system, where the hydrogel layer functions as the temperature-sensitive component. As the temperature changes, the conductive network within the hydrogel responds by varying its electrical resistance, mainly due to changes in ion movement and molecular interactions. This allows the system to monitor temperature in real time. When combined with other sensing layers it can simultaneously detect temperature, pressure, and proximity, making it well-suited for wearable applications such as sleep monitoring [234].

Recently, smart hydrogel-based sensors have been developed with multiple applications. Liu et al. (2024) developed a novel multifunctional self-sensing gradient hydrogel. Owing to its ultra-fast thermoresponsive actuation and high sensitivity, it has a high sensitivity of (GF ≈ 3.94), a fast response time of (~140 ms), and a high stretchability of up to ~600%. The hydrogel sensor can be used in wearable health monitoring, soft robotics, the human–machine interface, remote control systems (IoT), and smart sensing devices [236]. Moreover, hydrogel-based sensors used on wounds not only sense the temperature of the wound, but they can also offer a proper treatment plan. For instance, Jiang et al. (2025) synthesized a composite hydrogel, prepared through a simple one-step process with improved strength and adhesiveness and multifunctional properties. It is shown in Figure 7B that hydrogels work as dual-responsive wound dressings that combine temperature sensing with light-triggered treatment. The PNIPAM matrix allows the continuous, real-time monitoring of wound temperature, helping to detect inflammation while maintaining good contact with the skin. When exposed to near-infrared (NIR) light, the embedded nanoparticles produce heat and release nitric oxide (NO), which provides antibacterial and anti-inflammatory benefits. This system offers a smart and efficient way to monitor wound treatment, supporting faster and more effective healing [235]. Similarly, Hasallari et al. (2025) introduced a biocompatible hydrogel-based thermometer for high-sensitivity temperature monitoring via T_1_-weighted magnetic resonance imaging (MRI). In contrast to conventional aqueous Gd-complexes, the Fmoc-K_2_/Gd-1 hydrogel demonstrates a strong positive temperature relativity correlation, achieving a notable sensitivity (Δr_1_/°C) of 0.54 s^−1^/°C at 1 T (40 MHz). This work introduces a new class of injectable, responsive soft materials for precise MRI thermometry, with broad translational potential in thermal therapies and physiological monitoring. Also, the device retains structural and functional stability under physiological conditions and delivers reliable temperature reporting both in vitro and in vivo. Notably, subcutaneously injected hydrogel yields MRI-based thermal readouts that agree excellently with invasive thermometry [237]. These advances collectively demonstrate that hydrogel-based temperature sensors are evolving from simple threshold detectors toward multimodal, wirelessly integrated, and therapeutically coupled platforms capable of real-time physiological monitoring across diverse biomedical settings.

#### 5.1.3. Mechanical and Strain Deformation Sensing

Hydrogel-based mechanical and strain sensors have gained increasing attention as flexible sensing materials because of their soft, deformable networks, tunable conductivity, and excellent compatibility with wearable devices [238,239,240]. By integrating dynamic crosslinking, conductive nanomaterials, and multifunctional network architectures, recent studies have substantially improved the mechanical robustness, sensitivity, and practical applicability of these sensors [241,242]. For example, Zhou et al. (2026) developed a dual-network MXene-based hydrogel modified with polydopamine and silver nanoparticles, enabling high-performance strain sensing for handwriting recognition and wrist-movement monitoring [243]. In a related direction, Wang et al. (2025) reported a multimodal hydrogel-based M-PPT sensor that combined high sensitivity with pressure and temperature monitoring, demonstrating its utility in sleep-related physiological tracking [234]. Similarly, Zhou et al. (2026) fabricated a PEDOT: PSS-based conductive hydrogel with enhanced toughness, conductivity, and fatigue resistance, allowing the reliable detection of subtle physiological deformations such as vocal cord vibrations, breathing, and joint motion. Collectively, these studies demonstrate that hydrogel-based mechanical and strain sensors are evolving into versatile platforms for wearable health monitoring, motion analysis, and intelligent human–machine interaction [45]. Moreover, the multifunctional M-PPT organic hydrogel pressure sensor detects pressure through microstructure-assisted ionic capacitance changes, where pressure increases the hydrogel electrode contact area and promotes interfacial ion redistribution. Its fast response, low detection limit, stable dynamic output, and weak temperature interference make it promising for wearable pressure sensing and human–machine interaction [234]. Table 6 shows how hydrogel-based mechanical sensors combine high elongation, conductivity, gauge factor (GF), toughness, response time, and responsiveness to strain and other external stimuli. The strain sensing hydrogels, such as PEAAGG, TCPH, Casein-PAM, MXene/BC/PG-AA, PAAm-DA@CMC-MXene, PAAm/CMCs-Fe^3+^ DN, and PPTP, exhibit wide elongation ranges from about 290% to 4710%, tensile strengths up to 10.94 MPa, and gauge factors reaching 8.0, indicating their ability to detect both small and large body motions. Conductive fillers such as MXene, carbon-based components, and dynamic ionic networks improve sensitivity and signal stability. In comparison, CB-DNGH-20 is mainly designed for pressure sensing, showing a pressure sensitivity of 0.258 kPa^−1^ and a fast response time of 80 ms.

Recent studies demonstrate that hydrogel-based mechanical and pressure sensors are evolving into high-performance soft sensing platforms that integrate stretchability, conductivity, sensitivity, and fast response. Their ability to detect both subtle physiological signals and large human motions highlights their strong potential for wearable electronics, health monitoring, and human–machine interfaces [249,250,251].

#### 5.1.4. Molecular Detection and Biosensing

**Figure 8:**
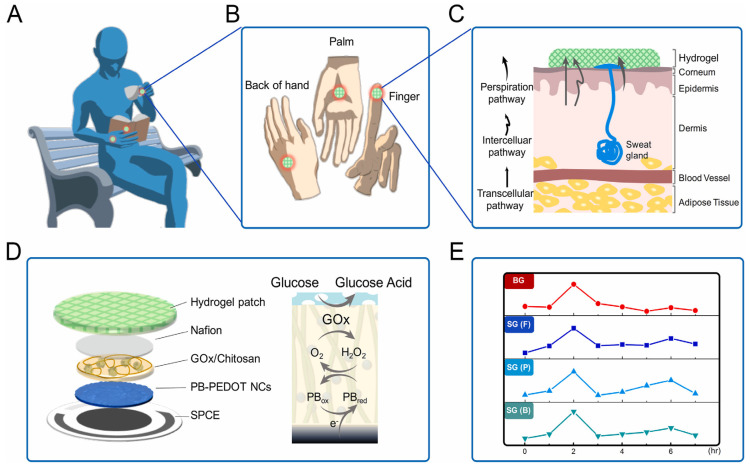
Schematic of design, mechanism, and usage of natural sweat sampling glucose sensor. (**A**) Hydrogel applied on skin. (**B**) Hydrogel applied on Palm, finger and back of the hand. (**C**) Sampling and sweat route to skin. (**D**) Multiple layers of hydrogel for sensing. (**E**) Signal detection [256]. Copyrights: images have been taken and reproduced from © 2022, Elsevier.

Hydrogel-based sensors have attracted significant attention for molecular detection and biosensing [60,84]. In biomedical fields, hydrogel-based sensors have been widely utilized for the detection of glucose [252], dopamine [61,253], lactate [254], uric acid, nucleic acids, proteins, and other clinically relevant biomarkers, supporting applications in disease diagnosis, wearable health monitoring, and the real-time analysis of physiological processes [199]. Molecule sensing is essential in the diagnosis of medical diseases and health monitoring. Different molecules are observed to monitor health and disease in patients. Among these target molecules, dopamine sensing remains challenging because of interference from structurally similar molecules, such as epinephrine, and the need to maintain signal stability under deformation. To address this, Liu et al. (2026) developed an AAM/CNT/MoS_2_ hydrogel-based electrochemical sensor with a low detection limit of 6.1 nM and stable performance under 50% strain over 15 stretch release cycles, highlighting its potential for real-time dopamine monitoring and neurological disorder diagnostics such as Parkinson’s disease, schizophrenia, and depression [61]. Recently, hydrogel-based sensors have been used for sweat analysis because sweat contains a broad range of biomolecules and electrolytes that reflect physiological and pathological states, making these platforms highly suitable for wearable, noninvasive, and real-time health monitoring [255]. Figure 8A–C shows that the noninvasive sweat glucose sensor is applied on the skin for the sampling of the sweat during normal activities in daily life. The sweat from the sweat gland is collected from the specific parts of the body, especially from the finger tips, palm, and back of the hand. The hydrogel patches provide continuous hydrophilic pathways from glands to the skin to collect glucose and other metabolites. Figure 8D shows that the collected sample is transferred to the multilayered electrochemical sensor with PB-PEDOT nanocomposites, glucose oxidase, and a Nafion protective layer. Glucose oxidase generates H_2_O_2_ proportional to glucose concentration, which is rapidly reduced by PB for electrochemical detection. The sensor enables hourly, long-term glucose monitoring, offering a convenient solution for routine glucose management (Figure 8E) [256].

Hydrogel-based sensors can be used to monitor cancer. Chen et al. (2025) developed a flexible wearable SERS hydrogel sensor for the noninvasive monitoring of lung cancer treatment effects via sweat biomarker analysis. The PVA-sucrose hydrogel absorbs sweat and protects Ag dendrite SERS substrates functionalized with five molecular receptors (4-MPBA, 4-MBA, 4-MPY, 2-NT, 4-ATP) for the multiplexed detection of glucose [87,89,257], pH, carbonyl compounds, and aldehydes. The sensor detects uric acid down to 5 × 10^−8^ M and creatinine to 10^−7^ M, with SERS stability exceeding eight days. Clinical validation on 12,617 spectra from 31 lung cancer patients achieved 89.7% accuracy in classifying treatment effects (progressive disease, partial response, no change) using Light GBM AI algorithms (AUC = 0.97). The sensor identified carbonyl biomarkers (I_1611_/I_1583_ ratio from 4-MPY) linked to diabetes complications and aldehyde signatures (1441 cm^−1^, 1073 cm^−1^) in squamous cell carcinoma monitoring, demonstrating the potential of personalized lung cancer treatment with comorbidities [89]. These applications collectively highlight how hydrogel-based molecular detection platforms are advancing from single-analyte sensors toward multiplexed, AI-assisted, and wearable systems capable of continuous, noninvasive biomarker monitoring across disease diagnosis and treatment assessment.

### 5.2. Hydrogel-Based Sensors in Environmental Monitoring

Beyond their applications in the biomedical domain, hydrogel-based sensors have gained significant importance in environmental monitoring. This section discusses hydrogel-based pH sensors, metal ion sensors, and humidity and gas sensors for environmental applications.

#### 5.2.1. Environmental pH Monitoring

Hydrogel-based pH sensors have emerged as versatile and reliable alternatives to conventional glass electrodes for environmental monitoring, offering rapid response, visual readout, mechanical flexibility, and stable operation under extreme pH conditions where traditional probes suffer from alkaline error, chemical deterioration, and fragility incompatible with continuous on-site deployment [147,231,258]. Addressing the critical limitation of conventional glass electrodes in extreme alkaline environments, Hosseinlou et al. (2024) developed chitosan-based hydrogel/BTB pH sensor, specifically designed for monitoring pH under extreme alkaline conditions (pH 10–14), where traditional glass electrodes suffer from “alkaline error” and chemical deterioration. The authors successfully applied the sensor to real samples including alkaline tap water, carbonate buffer, and ethanol amine buffer, demonstrating excellent recovery rates (94.09–108.94%) and good reproducibility. The sensor offers a dual detection method colorimetric (pH 11–14) and gravimetric (pH 10–14), making it suitable for accurate pH determination in highly alkaline aqueous solutions where conventional pH probes are unreliable [259]. Complementing this work, He et al. (2025) introduced a hydrogel for real-time environmental monitoring, specifically for the early warning of acid-base leakage accidents. The sensor responds within 4 s upon contact with acidic (pH 1–4) or alkaline (pH 11–14) environments. It can qualitatively and quantitatively identify different types of acids and bases through distinct electrical impedance signals. The authors proposed that this flexible hydrogel can be deployed on complex surfaces such as pipe joints and container walls to detect corrosive leaks and provide rapid warnings before environmental or human harm occurs [258]. Similarly, Güngör and Ozay (2022) developed a cationic hydrogel for rapid colorimetric pH sensing and successfully applied it to synthetic textile wastewater. The sensor determined wastewater pH within 60 s, outperforming glass electrode meters (150 s) and commercial pH strips (300 s). It exhibited a wide response range (pH 7–11), fast response (10–60 s), excellent reusability over five cycles, and maintained functionality in real samples including vinegar, juice, and bleach [231]. Thus, these findings confirm that hydrogel-based pH sensors are promising tools for real-time, on-site environmental water quality assessment, particularly in extreme pH conditions and complex industrial wastewater matrices.

#### 5.2.2. Metal Ion Detection

The metal ions, especially the heavy metal ions in various sources like soil and water, have caused devastating issues for aquatic life and human beings. The ions add to the supply chain of organisms and disrupt their life. Hydrogel-based sensors are used for metal ion detection to address critical environmental [260,261] and public health concerns associated with the heavy metal contamination of water supplies, food matrices, and occupational environments [262,263]. Cadmium is a toxic heavy metal that causes severe health problems in humans (kidney damage, bone disorders, and cancer, as it bioaccumulates, disrupting biological function and reproduction in organisms, and leads to long-term ecosystem imbalance. For cadmium detection, Wang et al. (2025) synthesized a temperature-responsive fluorescence hydrogel sensor by assembling a CdTe/CdS@dBSA quantum-dot probe into a PNIPAM network. The hydrogel provides dual adsorption (physical uptake by PNIPAM and coordination/chemisorption by dBSA), which enriches Cd^2+^ around the QDs and triggers chelation-enhanced fluorescence (CHEF). The platform operates as a dual-mode sensor, enabling quantitative Cd^2+^ detection through both fluorescence readout and smartphone-based RGB colorimetric analysis under UV light. This approach supports the portable, on-site monitoring of cadmium contamination in real samples such as food and water [264]. Further, hydrogel-based sensors are used to detect chloride ions as well. To detect the chloride ions a hydrogel-based colorimetric sweat sensor was developed by Tai et al. (2025) that allows the noninvasive detection of chloride (Cl^−^) by efficiently absorbing sweat. The sensor shows a linear response, with a detection limit of 0.56 mM and a working range of 20–100 mM, which is suitable for cystic fibrosis screening. The sensor remains stable for up to one month, making it practical for real-time wearable monitoring [122]. Chen et al. developed a dual-network hydrogel sensor for the highly specific and sensitive detection of Fe^3+^ ions in water. The sensor’s conductivity responded selectively to Fe^3+^ without interference from other metal ions or anions, achieving a minimum lower detection limit of 0.52 ppm and demonstrating excellent cyclic stability. The authors proposed that this hydrogel can be integrated with electronic devices at marine outfalls or sewage discharge points for real-time water quality monitoring, triggering an early warning when Fe^3+^ levels exceed the conductivity threshold of 0.16 S/m [265].

Table 7 reveals that the enlisted hydrogel-based metal ion sensors employ four principal transduction mechanisms, optical resonance shift, fluorescence quenching/turn-on, photonic structural color response, and ratiometric fluorescence, each offering distinct analytical advantages and limitations. Optofluidic microcavity sensors achieve the fastest response at 0.75 min for both Pb^2+^ and Hg^2+^ detection [260], establishing them as the most time-efficient platform, though their complex fabrication and instrument-dependent readout limit field deployment. Fluorescence-based systems dominate the table numerically, covering the broadest range of target ions including Co^2+^, Cu^2+^, Ni^2+^, Fe^3+^, Hg^2+^, Cr(VI), and Mn^2+^ [266,267], yet their response times span from 10 to 300 min, the widest variability in the table reflecting the fundamental trade-off between selectivity and speed inherent to probe-analyte binding kinetics. The quantum dot-doped tapered hydrogel waveguide achieves the ratiometric fluorescence sensing of Pb^2+^ within 1.5 min [268], combining speed with quantitative precision, making it the strongest candidate for point-of-care applications. Photonic structural color sensing via PNBC hydrogel responds within 5 min [269] and uniquely integrates detection with simultaneous removal, a dual functionality absent from all other entries. DNA-incorporating agarose hydrogels extend detection to physiologically relevant ions K^+^ and Hg^2+^ with intelligent image recognition within 90 min [270], though their biological probe dependency introduces storage and stability concerns. The CO_2_-responsive optical fiber platform [271] is the only entry exploiting a gas-triggered adsorption mechanism for Pb^2+^ detection, representing an entirely distinct sensing paradigm but lacking reported response time data, which limits direct performance comparison [260,261]. Critically, while all platforms demonstrate selectivity for their target ions, none simultaneously achieves a sub-minute response, naked-eye readout, multi-ion detection, and field-deployable fabrication, confirming that no single hydrogel-based metal ion sensing strategy is universally optimal. It is evident that hydrogel-based metal ion sensors are transitioning from laboratory tools toward portable, on-site, and naked-eye detection systems capable of addressing real-world environmental and public health monitoring demands.

#### 5.2.3. Humidity and Gas Sensing

Hydrogels, due to their inherent hydrophilicity and tailorable network architecture, exhibit rapid and sensitive responses to changes in relative humidity and gases, making them promising materials for wearable and flexible sensing applications [277]. Hydrogel-based humidity sensors primarily work through the moisture-induced swelling of the polymer network, whereby the absorption of water molecules changes the hydrogel’s thickness, volume, and ionic conductivity, enabling both optical and electrical transduction pathways [278,279]. Hydrogel-based humidity sensors can sense humidity effectively; for instance, a high-performance humidity sensor was synthesized through toxicant-free processing with exceptional performance, with a rapid response/recovery time (5.5/43.3 s), good repeatability, an ultra-high response value (9.3 × 10^5^), and a broad humidity detection range (0–97% relative humidity). Thus, the hydrogel can be applied for real-time breath monitoring and non-contact human–machine interfaces. The relative humidity of the hydrogel at 11% chemisorption is lower, and as the relative humidity increases, the water absorption and the conductivity also increase. The Grotthuss chain reaction (H_2_O + H_3_O^+^ → H_3_O^+^ + H_2_O) enables charge (H^+^) to transfer, resulting in an increase in conductivity [280].

Hydrogel-based gas sensors have been developed for the detection of environmentally and medically relevant gases, including CO_2_ [271], NH_3_ [281,282,283], NO_2_ [283], H_2_S [284], and volatile organic compounds (VOCs) [285]. The high-water content of hydrogels facilitates the dissolution and chemical reaction of water-soluble gases, providing inherent gas selectivity based on aqueous-phase chemistry [271,278,286]. Thus, the hydrogel-based gas sensors have attracted considerable interest because their high water content, soft three-dimensional networks, and abundant functional groups enable the efficient absorption of water-soluble gases and strong physicochemical interactions with analytes [278,287]. These features make hydrogels particularly promising for applications such as environmental monitoring, food quality assessment, healthcare diagnostics, and wearable breath analysis [288]. Ammonia and nitrogen dioxide are monitored in industries to avoid critical health issues in workers and chemical processes. Wu et al. (2015) synthesized a hydrogel-based sensor that could monitor ammonia and nitrogen dioxide in a range of concentrations of 200 ppm and 20 ppm. The sensor can be used to monitor these gases at various industrial or other premises [289]. Similarly, Zhi et al. (2020) formulated a self-responsive sensing mechanism-based hydrogel that exhibits rapid responses to NO_2_ and NH_3_. The sensor shows excellent conductivity and mechanical properties. Thus, the sensing mechanism is an alternative for gas sensing [283]. However, despite their promise for wearable and flexible sensing applications, hydrogel-based humidity and gas sensors face critical limitations that hinder practical deployment. Their high water content, essential for ionic conductivity and gas dissolution, renders them prone to rapid dehydration, leading to signal drift, mechanical embrittlement, and diminished sensitivity over time [251]. Additionally, cross-sensitivity to ambient humidity and temperature fluctuations complicates accurate target-gas differentiation. Resolving these limitations requires organohydrogel formulations incorporating glycerol or ionic liquids to suppress dehydration [161], molecularly imprinted polymer networks to improve analyte specificity [278], and self-powered device architectures that eliminate external power requirements while maintaining sensitivity across humidity ranges [290]. Across both biomedical and environmental domains, hydrogel-based sensors have demonstrated remarkable versatility, detecting pH, temperature, mechanical strain, molecular biomarkers, metal ions, humidity, and gases within a single material platform. However, translating these laboratory demonstrations into clinically and industrially deployable systems requires addressing persistent challenges in long-term stability, selectivity, scalability, and device integration, as discussed in the following section.

## 6. Future Outlooks

Despite remarkable advances, translating hydrogel-based sensors into clinically and industrially deployable systems requires resolving four interconnected challenges: long-term environmental stability, multimodal signal integration, scalable manufacturing, and intelligent data processing. Environmental instability remains the most critical barrier. Dehydration, interfacial delamination, and swelling drift continue to limit real-world deployment, particularly in implantable and gas-sensing configurations [38,85]. Future designs must systematically embed antifouling surface chemistries, dynamic encapsulation, and water-retention strategies as mandatory design criteria rather than post-fabrication corrections. Anti-freezing organohydrogel formulations [160] and polyelectrolyte-based networks [161] represent promising but not yet universally applicable solutions. Moreover, multimodal sensing platforms capable of simultaneously detecting mechanical, thermal, chemical, and biochemical stimuli represent the next frontier. Self-powered thermogalvanic hydrogels achieving a thermopower of 1.44 mV/K [219] and piezoionic systems reaching GF of 1242 [222] demonstrate the potential of integrated self-powered architectures, though realizing independent signal channels within a single network without crosstalk remains an open engineering challenge [210,216].

Scalable manufacturing and standardized evaluation protocols are equally critical. Batch-to-batch variability arising from UV curing and narrow gelation windows limits reproducibility [129], while 3D printing [149,251], and digital light processing [169] offer emerging solutions. Standardized metrics covering sensitivity, drift, cycling stability, and shelf life currently absent across the field are essential for regulatory translation [85]. Machine learning integration represents an underdeveloped but transformative frontier. AI-assisted SERS analysis has achieved 89.7% accuracy in lung cancer treatment classification [89], and machine learning-enabled thermogalvanic sensors have demonstrated multi-signal decoupling [219]. Expanding these approaches toward real-time drift correction and predictive diagnostics particularly within MXene and MOF composite platforms [88,215] will be decisive for clinical translation. Simultaneously, fully bio-based systems such as enzymatically crosslinked gelatin organohydrogels [156] and recyclable light-triggered networks [245] point toward sustainable circular design strategies. The future of hydrogel-based sensors lies in the convergence of robust material engineering, intelligent fabrication, and data-driven analytics.

## 7. Conclusions

This review has provided a systematically integrated analysis of hydrogel-based sensors, linking polymer composition, crosslinking strategy, fabrication method, transduction mechanism, and sensing application within a unified design-to-performance framework. Natural polymers offer biocompatibility and abundant reactive groups but are inherently limited by low conductivity, poor mechanical robustness, and environmental instability constraints rooted in molecular architecture rather than incidental formulation choices. Hybrid network designs consistently outperform single-polymer systems, confirming that complementary chemistry is the necessary direction. Synthetic polymers provide superior tunability but introduce persistent stretchability–sensitivity trade-offs and scalability constraints, with carboxyl group density, network topology, and filler dispersion homogeneity emerging as the primary molecular determinants of gauge factor, hysteresis, and environmental stability. Among fabrication strategies, chemical crosslinking provides structural permanence, physical crosslinking enables dynamic reversibility, and composite fabrication synergistically enhances electrical performance through percolative nanofiller integration. No single strategy is universally sufficient; their intelligent combination governs transduction efficiency and operational stability in ways that cannot be recovered by downstream device engineering.

Across transduction mechanisms, piezoresistive and piezoionic sensing excel in mechanical responsiveness, capacitive sensing provides broad-range pressure linearity, thermogalvanic sensing uniquely enables self-powered operation, and electrochemical, colorimetric, and fluorescence sensing deliver nanomolar chemical precision. Applications spanning wound monitoring, fever detection, strain sensing, sweat biomarker analysis, heavy metal detection, and gas sensing collectively demonstrate platform versatility across biomedical and environmental domains. Despite these advances, dehydration-induced drift, long-term instability, limited selectivity in complex matrices, interfacial failure, and the absence of standardized evaluation protocols remain the primary barriers to clinical and industrial translation. Overcoming these challenges will require multifunctional network designs integrating dynamic crosslinking, conductive nanofillers, antifouling interfaces, and machine learning-assisted signal processing within reproducible fabrication workflows. Hydrogel-based sensors represent a convergent platform at the intersection of polymer chemistry, nanotechnology, flexible electronics, and intelligent analytics, uniquely positioned to enable next-generation wearable, implantable, and environmental sensing technologies, provided the field transitions from performance demonstration to standardized, clinically validated, and scalable manufactured device systems.

## Figures and Tables

**Table 1:** Summary of natural polymer-based hydrogel sensors: Key properties, performance metrics, and inherent limitations.

Natural Polymer Based Hydrogel	Sensor Type	Key Properties	Limitations	Ref.
Cellulose Nanofibers (CNF) + PVA + Polypyrrole (PPy)	Strain sensor	GF = 2.84; conductivity 0.034 S/m; stress ~0.65 MPa; strain ~301%; anti-freezing (−18 °C); 300 cycles	Limited cycling (300 cycles); PPy self-aggregation risk	[123]
Cellulose Nanocrystals (CNC) + HA/PAA	Antifreezing strain sensor	GF = 0.8; self-healing 86.7%; response/recovery 351.8/352.4 ms; anti-freezing (−54 °C); >50 cycles	Very low GF; very few cycles (>50 only)	[111]
TEMPO-oxidized Cellulose Nanofibers (TOCN) + PPy + Glycerol	Antifreezing strain sensor	GF = 2.41; response 100 ms; anti-freezing (−45 °C); 10 cycles	Only 10 cycles; glycerol biodegradability unclear	[112]
Cellulose Nanocrystals (CNC) + PANI	Strain sensor	GF = 1.68; response 96 ms; stable −60 °C to 80 °C; >10,000 cycles; self-healing; adhesive	Moderate GF value	[124]
Hydroxypropyl Cellulose (HPC) + black conductive hydrogel	Dual-mode strain sensor	GF = 2.99 (0–150%), 4.24 (150–300%); mechanochromic contrast 4.92; absorbs >88% visible light; >4400 cycles	Complex physical + chemical crosslinking	[125]
Cellulose Nanofibers (CNF) + PVA	Sweat biomarker sensor (urea)	Detection limit 0.19 uM; elongation 2013.5%; dual colorimetric + fluorescence; clinical range 5–40 mM	Limited to urea detection only	[126]
Bacterial Cellulose (BC) + Carboxymethyl Cellulose (CMC) + Chitosan	Sweat biomarker sensor (Cl^−^ + glucose)	Tensile force 4.16 N; Cl^−^ range 20–100 mM (limit 0.56 mM); glucose range 6.25–500 uM (limit 0.1 uM); dual simultaneous detection	Colorimetric readout only; requires visual or instrument-based analysis	[122]
Bacterial Cellulose (BC)	Sweat biomarker sensor (alcohol)	Detects 0–30 mM alcohol; self-adhesive; stable under bending/stretching; biodegradable; no skin irritation (3 h)	Inherent hydrophilicity required structural modification (sandwich design) to overcome	[127]
Sodium Alginate (SA) + Polyacrylamide (PAM) double-network hydrogel	Flexible strain sensor	Stress 0.52 MPa; sensing range 300%; anti-freezing (−20 °C); self-adhesion 9.5 kPa; TENG output 63.7 V; switchable conductive/adhesive/sensing modules	Fe^3+^ coordination gives weakest adhesion; EDTA recycling adds processing step	[114]
Sodium Alginate (SA) + Polyacrylamide (PAM) + Gelatin + Ca^2+^ + LiCl	Flexible strain/pressure sensor	GF = 1.07; pressure sensitivity 0.0107 kPa^−1^; strain 1500%; conductivity 1.5 S/m; transparency ~75%; anti-freezing (−20 °C); >2000 cycles; response 420 ms	Low conductivity without LiCl; excessive Ca^2+^ reduces elongation sharply	[113]
Gelatin + Dialdehyde TEMPO-oxidized Nanofibrillated Cellulose (DATNFC) + Fe^3+^	Multifunctional strain sensor	GF = 2.24; compressive strength 1310 kPa; tensile strength 164.7 kPa; strain 990.7%; conductivity 0.0227 S/m; self-healing; recyclable; 150 cycles; response 400 ms	Partial self-healing recovery (tensile 36.7%, modulus 45.3%)	[115]
Fish Gelatin (FG) + Acrylamide (AM) + Cellulose Nanofiber (CNF) + Ag Nanoparticles (AgNPs) + KCl	Strain + self-powered pressure sensor	GF = 4; strain 2600%; self-adhesion 14 kPa; antibacterial (*E. coli* + *S. aureus*); TENG Voc = 232 V; wound healing rate 95.43%; 500 cycles (strain), 5000 cycles (TENG)	Water retention limited (~50% after 13 h); poor elasticity of pure fish gelatin	[116]
Chitosan (CS) + Polyacrylic Acid (PAA) + Tannic Acid (TA) + Fe^3+^	Multifunctional strain/EMG/ECG sensor	GF = 1.84–2.72; stress 91 kPa; strain 1109%; conductivity 0.93 S/m; self-healing 89.2% (conductivity), 95.6% (strain); adhesion 327 kPa (paper), 133 kPa (pigskin); response 670 ms; ECG (81 bpm) + EMG	Moderate stress (91 kPa); slight resistance drift over 100 cycles	[117]
Chitosan (CS) + Polyacrylic Acid (PAA) + DOPA + Zn^2+^	Wearable strain sensor	GF = 2.92–25.18; stress 164 kPa; strain 1100%; conductivity 0.88 S/m; adhesion 30 kPa (aluminum), 23.9 kPa (pigskin)	No self-healing; low skin adhesion (23.9 kPa)	[118]
Chitosan (CS) + Polyacrylamide (PAM) + Al^3+^	Wearable strain sensor	GF = 0.9–3.0; stress 150 kPa; strain 1040%; conductivity 5.01 S/m; adhesion 17.1 kPa (glass), 29.9 kPa (wood)	No self-healing; low adhesion strength	[119]
Starch + PVA + [Emim]Ac + AlCl_3_	Multifunctional wearable sensor (strain/pressure/temperature/underwater)	GF = 5.93 (350–400%); conductivity 2.75 S/m; anti-freezing (−20 °C); antibacterial (*E. coli* + *S. aureus*); anti-swelling 52.8%; temperature sensing RT-70 °C	Poor mechanical properties without additives; water content trade-off vs. conductivity	[120]
High-amylose starch + CaCl_2_ + Glycerol	Self-powered wearable sensor	GF = 1.39 (5–170%); pressure sensitivity 1.5371 kPa^−1^; strain 208%; battery voltage 0.81 V; anti-freezing (−45 °C); self-healing at 55 °C; biodegradable >85% in 40 days	SP sensor current decreases over 1000 cycles; poor mechanical strength vs. synthetic polymers	[121]

**Table 2:** Synthetic polymers used in hydrogel-based strain sensors: Key properties, performance metrics, and limitations.

Polymer (Abbr.)	Type of Sensor	Key Properties	Limitations	Ref.
PVA	Soft wearable strain sensor	Stretchability: 1000%; Self-healing: 3 s (95–98% efficiency); Response: 414 ms; Recovery: 460 ms (Milliseconds); GF: 1.94 (0–100% strain); Cyclic stability: >1000 cycles at 20% strain	PVA concentration critical (0.2 g optimized); Self-healing degrades after >2 cycles; Requires UV curing; Oxygen sensitive during; Requires glycerol to prevent dehydration	[140]
AMPS	Strain sensor (wearables)	Sulfonated monomer boosts ionic conductivity; Enhances mechanical resilience for 1000% stretchability; Enables repeatable signals over >1000 cycles	Excessive AMPS reduce conductivity (aggregation) Requires precise concentration control	[140]
AM/pAAm	Stretchable strain/haptic sensors	Stretchability: ~3000%; Conductivity: 0.15–0.44 S m^−^; Gauge factor (GF): 0.52 (0–172%), 2.29 (172–502%), 3.74 (502–834%); Self-healing: ≈100% recovery (host–guest and H-bonding); Response time: ≈0.2 s; Cyclic stability: >200 cycles (≈96% retention)	Requires β-CD and AETAc for SPN formation; Optimal balance requires 1:1 AAm/AETAc ratio; Conductivity lower than PEDOT:PSS (~40 S/cm); Transparency decreases with thickness; Requires one-pot synthesis at 60 °C for 4 h	[140,141]
PNIPAM	Flexible strain sensor (solvent environments)	Elongation; 2512%; Tensile strength: 29 kPa; GF range: 2.14–4.96; Response time: 64 ms; Recovery time: 202 ms; Cyclic stability: 4000 cycles; Adhesion; (glass): 29.17 g; Solvent stability: >400 days; Compressive strength: 187 kPa	Requires BIS, GO, and PEDOT: PSS for stability; Conductivity depends on PEDOT: PSS content; Swelling occurs in water/ethanol/acid (network intact); Lower GF than some nanocomposite sensors	[142]
PEDOT:PSS	Conductive hydrogel strain sensors	Conductive polymer integrated into IPN with PNIPAM; Forms fully polymeric conductive hydrogels with high stretchability (2512%); Rapid recovery (202 ms); Stable strain sensing without metallic fillers; Conductivity increases with PEDOT:PSS content	Conductivity depends on content; Requires BIS and GO for chemical stability; Lower conductivity than pure PEDOT: PSS due to PNIPAM matrix	[142,143]
poly (HEAA-co-SBAA)	Self-adhesive conductive strain sensors	Stretchability: 4000–5000%; Tensile strength: ~0.5 MPa; Self-healing: <3 min; Toughness recovery: 70–80% in 5 min; Interfacial toughness: ~1700 J m^−2^; GF: ~2.0; Conductivity: 0.625 S/m; Antifouling; Biocompatible (91–95% cell viability)	Requires PEDOT:PSS for conductivity; Gauge factor lower than some nanocomposite sensors; Acidic PEDOT:PSS slightly reduces cell viability	[143]
PAA	Strain sensor (wearable)	Strain-stiffening (~10.5 MPa); self-healing (98.5%); low hysteresis (η ≈ 0.2); GF = 1.66 (0–200%); response: 0.21/0.28 s; detects 0.5–10% strains Cyclic stability: 200 cycles at 50% strain	Requires LiCl; hysteresis at >200% strain Requires UV curing (λ = 365 nm (nanometer), 8 W, 0.5 h)	[137]
P(SBMA-co-AAm)	strain sensors	Elongation: 1353%; Conductivity: 0.15 S/m; Tensile strength: 50.6–146.1 kPa; Self-healing: 85.05% in 30 min; Biocompatible (>97%); Self-cleaning	Hysteresis; ionic bonds break under deformation; requires alginate for mechanical reinforcement	[144]
pAAm/carrageenan	strain multi-stimuli sensors	Strain range: 0.5–950%; GF: 6 (250–400%); Recovery: 0.3 s; Self-healing: 170% strain, 96.25% electrical recovery	Requires organohydrogel formation for extreme stability; sensitivity varies with water content	[145]

**Table 3:** Comparison of chemical crosslinking methods for hydrogel-based sensors: Bond type, reversibility, gelation conditions, advantages, and limitations.

Crosslinking Type	Bond Type	Reversibility	Typical Gelation Condition	Key Advantage	Main Limitation	Ref.
Free radical polymerization	Covalent	No	Thermal (80 °C)	Strong, stable network	Poor self-healing	[179]
Schiff base	Dynamic covalent	Yes	Mild pH, aqueous	Self-healing	pH-sensitive	[180]
Boronic ester	Dynamic covalent	Yes	Alkaline/aqueous	Fast self-healing	pH dependent	[181]
Thiol-ene click	Covalent	No	UV/photo initiator	Fast, efficient gelation	Functional precursors needed	[182,183]
Carbodiimide coupling	Covalent	No	EDC/NHS, mild pH	Strong bioconjugation	Multistep process	[128,184]
Enzymatic crosslinking	Covalent	No	Physiological conditions	Excellent biocompatibility	Slower reaction	[174]
Photo-crosslinking	Covalent	No	UV/visible light	Spatial control, rapid curing	Light penetration limits	[185]

**Table 4:** Physical crosslinking mechanisms in hydrogel-based sensors: Interactions, reversibility, advantages, and limitations.

Crosslinking Type	Interactions	Reversibility	Key Advantages	Main Limitations	Ref.
Freeze–thaw induced crystallization	PVA chain alignment into crystalline domains	Partially reversible (melts above ~60 °C)	No chemical crosslinkers; 346% stretchability; 1000-cycle stability at 30% strain	Requires 4 freeze–thaw cycles; network temperature-sensitive	[193]
	PVA chain alignment into crystalline domains (ice crystal template)	Partially reversible	Creates 3D porous microstructure; enhances mechanical strength	Requires 3 freeze–thaw cycles; temperature-sensitive	[189]
Ionic coordination	Ca^2+^ coordination with –COO^−^ of sodium alginate (egg-box junctions)	Reversible (via EDTA)	Rapid gelation (2 h in 0.2 M CaCl_2_); conductivity 0.53 S/m; SNR 18.2 dB for ECG	Potential Ca^2+^ leaching in physiological fluids	[193]
Hydrogen bonding	Phenolic –OH of TA with –OH of PVA, –OH of CNC, and –COOH of MWCNT-COOH	Fully reversible (dynamic break/reform)	High stretchability (600%); tensile strength 1.76 MPa; self-healing 97% (50 min); adhesion 27.42 kPa (fabric)	TA concentration critical (optimal 40%); requires immersion step	[189]
	Between SA and Amm (N-H. O=C); confirmed by FTIR red shift (3353 → 3333 cm^−1^)	Fully reversible	Enhances mechanical properties; contributes to energy dissipation	Weakens at high strain	[191]
Hydrophobic association	LMA chains aggregated in SDS micelles; dynamic dissociation conjugation	Fully reversible	Toughness 1.44 MJ/m^3^; stretchability 1021%; fracture stress 345 kPa; energy dissipation via chain sliding	Requires SDS (optimal 3 wt%); higher SDS causes phase separation	[190]
	LM chains aggregated in SDS micelles; dynamic dissociation under stress	Fully reversible	High stretchability (1400%); fracture stress 1254 kPa; low hysteresis (9 kJ/m^3^ 1st cycle)	Requires SDS and NaCl; optimal LM 15% (SLA3)	[191]
Biopolymer self-assembly	π–π stacking, hydrogen bonding, hydrophobic interactions, ionic interactions	Partially reversible	No additives/cross-linkers needed; high conductivity; large surface area; 3D porous network	Lower mechanical strength than covalently cross-linked hydrogels	[192]

**Table 5:** Comparative analysis of transduction mechanisms in hydrogel-based sensors: Sensitivity, response time, and detection limits.

Sensing Mechanism	Stimulus	Output Signal	Typical Sensitivity/Detection Range	Response Time	LOD	Ref.
Piezoresistive	Strain	ΔR/R_0_	2.23 kPa^−1^ (5–22 kPa)	Response 70 ms; recovery 100 ms at 25 kPa		[149]
	Strain	ΔR/R_0_	GF 2.27 (0–55% strain, R^2^ = 0.999); 25.76 (400–600% strain)			[150]
	Pressure	ΔR/R_0_	Maximum sensitivity: 2.27 kPa^−1^ (0–0.5 kPa); 0.18 kPa^−1^ (0.5–2 kPa); 0.021 kPa^−1^ (2–5 kPa)	Response: 0.18 s; Recovery: 0.17 s	9.0 Pa	[151]
Resistive (Ionic Conductive)	Strain	ΔR/R_0_	GF = 0.8 (0–550% strain); GF = 2.9 (550–1585% strain); Detection limit: 0.1% strain; Wide strain range: 0.1–1585%	123 ms (response); 197 ms (recovery)	0.1% strain	[217]
Capacitive	Mechanical pressure/proximity	ΔC/C_0_	Linear response 1–40 kPa (R^2^ = 0.99) and 400–1500 kPa (R^2^ = 0.98); sensitivity adjustable via dielectric layer gas volume	100 ms	Human motion detectable	[149]
	Ammonium (NH_4_^+^) conc. in water	Capacitance change (ΔC)	20% capacitance increase within 200 s at 3 µM; plateau above 1 mM	200 s (20% change)	3 µM (lowest tested concentration)	[208]
	Pressure	Relative Capacitance Change (RCC = (C − C_0_)/C_0_)	49.12 MPa^−1^ (0–0.1 MPa); 17.96 MPa^−1^ (0.1–0.3 MPa); 10.03 MPa^−1^ (0.3–0.6 MPa); 4.11 MPa^−1^ (0.6–1 MPa)			[209]
Thermogalvanic effect (general)	Temperature gradient (ΔT)	Open-circuit voltage	S (typical): 1–4 mV K^−1^ for Fe(CN)_6_^4−^/^3−^; up to 17 mV K^−1^ for optimized gelatin-based systems	Seconds to minutes	Depends on ΔT and redox couple	[218]
Strain–thermal coupling/Thermogalvanic	Strain + Temperature gradient (simultaneous coupling)	Open-circuit voltage (V)—self-generated	Sensitivity (thermopower): 1.06 mV K^−1^ (0% strain); 1.44 mV K^−1^ (100% strain) Detection range: Strain up to 200%, ΔT up to ~10 K	Strain: 0.41 s (1 Hz); 1.32 s (0.5 Hz); Thermal: 0.39 s (brief contact)		[219]
Electrochemical	Glucose, Lactate, Uric acid, H_2_O_2_, Alcohol	Current (µA)	Glucose: 340.1 µA mM^−1^ cm^−2^ (PEDOT:PSS/PB/GOx); Lactate: 35.3 µA mM^−1^ cm^−2^; Uric acid: 0.875 µA µM^−1^ cm^−2^; Ethanol: 0.362 µA mM^−1^	<6 s–<15 s	Glucose: 0.85 µM; Uric acid: <1.2 µM; Lactate: ~4 µM	[220]
Colorimetric	Chemical analytes—H_2_S gas, pH, heavy metals	Color change ΔE; RGB ratio	H_2_S: detection range 0.2–100 ppm; R^2^ = 99.76%; sensitivity 6.203–6.257	Response 10 s; recovery 32 s; total detection <1 min	0.026 ppm (theoretical); 0.2 ppm (experimental)	[195]
Ratiometric Fluorescence	Chemical analytes; antibiotics, metal ions, pH, glucose	Optical signal	Doxycycline: LOD 36.6 nanomolar(nM) (solution), 53.6 nM (hydrogel), 43.1 nM (smartphone platform); linear range 0–28.0 μM; R^2^ = 0.981–0.992	2 s reaction; complete within 10 s	36.6 nM (solution); 53.6–62.5 nM (real samples)	[221]
Piezoionic	Mechanical strain/bending	Self-powered current (nA)	GF = 1242 at 3.12% strain; R^2^ = 0.997; piezoionic coefficient 1.85 nA/Pa; Young’s modulus ~45 kPa	Response 40 ms; decay 90 ms	Subtle forces at ~45 kPa	[222]

**Table 6:** Mechanical and sensing properties of representative hydrogel-based strain and pressure sensors.

Hydrogel Name	Tensile Strength (MPa)	Elongation (%)	Toughness (kJ/m^3^)	Conductivity (S/m)	Gauge Factor (GF)	Response Time (ms)	Stimuli Responsive/Sensor	Ref.
PEAAGG Hydrogel	10.94	355	16.65 × 10^3^	0.15	0.94 (0–50%), 1.54 (50–125%)	235/222	Strain Sensor (Strain, Ion, Temperature responsive)	[244]
TCPH Hydrogel	0.33	1500	2040.76	1.89	2.26–6.5	176	Strain Senso Recyclable light, Strain, Enzyme res	[245]
Casein-PAM Hydrogel	>0.47	4710	—	Suitable for sensing	—	105	Strain Sensor	[246]
MXene/BC/PG-AA Hydrogel	0.152–0.185	862	—	Increases with MXene	0.36 (0–150%), 0.21 (150–430%), 1.28 (430–640%)	<150	Strain Sensor (Strain-responsive)	[247]
PAAm-DA@CMC-MXene Hydrogel	0.51	1100	5.374 × 10^3^	0.072	3.6 (0–250%), 6.3 (300–600%), 8.0 (650–800%)	102	Strain Sensor (Strain-responsive)	[248]
PAAm/CMCs-Fe^3+^ DN Hydrogel	0.44	715	1.658 × 10^3^	3.1	0.43 (0–200%), 0.8 (200–400%), 1.15 (400–700%)	—	Strain Sensor (Strain-responsive)	[249]
PPTP Hydrogel	2.6155	290	3.8 × 10^3^	7	3.75	125	Strain Sensor (Strain-responsive) + ECG Electrode + Information Encryption	[250]
CB-DNGH-20	0.68 ± 0.06	500	-	~0.1–0.25	0.258 kPa^−1^	80	Pressure sensor	[251]

**Table 7:** Summary of hydrogel-based sensors for metal ion detection: Target ions, response time, sensing mechanisms, and applications.

Hydrogel	Ion	t_res (min)	Sensing Mechanism	Application	Ref.
Hydrogel Optofluidic Microcavity	Pb^2+^	0.75	Optical resonance wavelength shift	Chinese herbal medicine screening	[260]
	Hg^2+^	0.75	Optical resonance wavelength shift	Chinese herbal medicine screening	
SA/PAM@MOF-Eu hydrogel	Co^2+^	10	Fluorescence quenching response	Environmental heavy metal monitoring	[266]
	Cu^2+^	10	Fluorescence quenching response	Environmental heavy metal monitoring	
	Ni^2+^	10	Fluorescence quenching response	Environmental heavy metal monitoring	
PNBC photonic hydrogel	Pb^2+^	5	Photonic structural color response	Lead ion detection and removal in water	[269]
Polyvinyl alcohol hydrogel AIE film	Hg^2+^	30	Fluorescent turn-on response	Environmental water mercury analysis	[272]
Fluorescent nanocellulose hydrogel	Fe^3+^	105	Fluorescence quenching response	Heavy metal detection and removal in water	[267]
	Pb^2+^	105	Adsorption-assisted fluorescent hydrogel response	Heavy metal detection and removal in water	
N, P-CDs@CMC/PEI composite hydrogel	Hg^2+^	300	Fluorescence quenching response	Toxic heavy metal detection and capture in water	[273]
	Fe^3+^	—	Fluorescence quenching response	Toxic heavy metal detection in water	
	Cr(VI)	300	Adsorption-coupled fluorescence platform	Toxic heavy metal capture in water	
Quantum dots-doped tapered hydrogel waveguide	Pb^2+^	1.5	Ratiometric fluorescence sensing	Point-of-care lead ion detection	[268]
Agar hydrogel with calcium-selective organosilica nanoparticles	Ca^2+^	2	Distance-based exhaustive colorimetric sensing	Calcium detection in blood and serum	[274]
DNA-incorporating agarose hydrogel DNA-incorporating agarose hydrogel	K^+^	90	Fluorescent DNA probe response with intelligent image recognition	On-site potassium detection in water and serum	[270]
	Hg^2+^	90	Fluorescent DNA probe response with intelligent image recognition	On-site mercury detection in serum and lake water	
HB-Alg/Gel@WTR-CDs hydrogel beads	Cr^6+^	10–15	Stimuli-responsive fluorescent quenching response	On-site naked-eye detection in water and environmental remediation	[275]
	Mn^7+^	10–15	Stimuli-responsive fluorescent quenching response	On-site naked-eye detection in water and environmental remediation	
CO_2_-responsive P(DMAEMA-co-HEMA)/CS hydrogel-functionalized optical fiber	Pb^2+^		Optical fiber interference spectrum shift via CO_2_-triggered adsorption/desorption	Optical fiber interference spectrum shift via CO_2_-triggered adsorption/desorption	[276]

## Data Availability

No new data were created or analyzed in this study. Data sharing is not applicable to this article.

## Abbreviations

The following abbreviations are used in this manuscript:

PVA	Polyvinyl Alcohol
PAA	Polyacrylic Acid
PAM	Polyacrylamide
PEG	Polyethylene Glycol
Ge	Gelatin
PNIPAM	Poly (N-isopropylacrylamide)
PDMS	Polydimethylsiloxane
P(DMAEMA-co-HEMA)	Poly(Dimethylaminoethyl Methacrylate-co-Hydroxyethyl Methacrylate)
CMC/CMCs	Carboxymethyl Cellulose/Carboxymethyl Chitosan
BC	Bacterial Cellulose
CS	Chitosan
SA	Sodium Alginate
TA	Tannic Acid
PG	Propylene Glycol
DA	Dopamine
DA@CMC	Dopamine-grafted Carboxymethyl Cellulose
PPTP	PVA-Pectin-TA-CaCl_2_/NaCl Hydrogel
APS	Ammonium Persulfate
MBAA/MBA/BIS	N,N′-Methylenebisacrylamide
TEMED	Tetramethylethylenediamine
DEAP	2,2-Diethoxyacetophenone
UV2959	2-Hydroxy-4′-(2-hydroxyethoxy)-2-methylpropiophenone
DTT	Dithiothreitol
VC	Vitamin C (Ascorbic Acid)
NSD	Nano-Silicon Dioxide
BP	Benzophenone
AA	Acrylic Acid
AAm	Acrylamide
AM	Acrylamide
AMPS	2-Acrylamido-2-Methylpropane Sulfonic Acid
g-C_3_N_4_	Graphitic Carbon Nitride
MXene (Ti_3_C_2_T_x_)	Titanium Carbide
MoS_2_	Molybdenum Disulfide
ZnO	Zinc Oxide
FeCl_3_	Ferric Chloride
CaCl_2_	Calcium Chloride
NaCl	Sodium Chloride
Cu(NO_3_)_2_	Copper(II) Nitrate
GF	Gauge Factor
LOD	Limit of Detection
RT	Room Temperature
NIR	Near-Infrared
UV	Ultraviolet
DN	Double Network
OFS	Optical Fiber Sensor
SERS	Surface enhanced Raman scattering
4-MBA	4-mercaptobenzoic acid
4-MPBA	4-mercaptophenylboronic acid
ECG	Electrocardiogram
EOL	End-of-Life
t_res	Response Time
CDPAP	Collagen-based multifunctional hydrogel (Collagen, Dialdehyde carboxymethyl cellulose, Polyacrylic acid, AlCl_3_, 1,3-Propanediol
DCMC	Dialdehyde carboxymethyl cellulose
BIS	N,N′-methylenebis(acrylamide)
GO	Graphene Oxide
MWNT	Multi-Walled Carbon Nanotubes
pAAm/carrageenan	Polyacrylamide/carrageenan double network
P(SBMA-co-AAm)	Poly(2-(methacryloyloxy)ethyl) dimethyl-(3-sulfopropyl) ammonium hydroxide-co-acrylamide
Poly(HEAA-co-SBAA)	Poly(2-hydroxyethyl acrylamide)-co-sulfobetaine acrylate
sc–PEG-PLA	Stereocomplex Poly(ethylene glycol)-b-poly (lactic acid)
PEDOT:PSS	Poly(3,4-ethylenedioxythiophene): polystyrenesulfonate
4-MPY	4-Mercaptopyridine
2-NT	2-Naphthalenethiol
4-ATP	4-Aminothiophenol
AI	Artificial Intelligence
LGB	Light Gradient Boosting Machine (Light GBM)
AUC	Area Under the Curve (Receiver Operating Characteristic curve)
PD	Progressive Disease
PR	Partial Response
NC	No Change
I_1611_/I_1583_	Ratio of Raman peak intensities
ECHs	Electronically Conductive Hydrogels

## References

[1] Setti Sudharsan M., Selvam L., Mani H., Pazhamalai V., Nattanmai Mothilal H., Asaithambi P. (2026). Unlocking the Potential of Hydrogel Microspheres for Sustainable Environmental Remediation. Pure Appl. Chem..

[2] Anjumnisha S., Prasad E. Hydrogel Market Size & Growth Share|Forecast Report, 2033. https://www.alliedmarketresearch.com/hydrogel-market.

[3] Dev K.G., Pal M. (2026). Challenges and Future Perspectives of Hydrogels in Wastewater Treatment. Applications of Hydrogels in Modern Wastewater Treatment.

[4] Shen C., Wang Y., Yuan P., Wei J., Bao J., Li Z. (2026). Conductive Hydrogels in Biomedical Engineering: Recent Advances and a Comprehensive Review. Gels.

[5] Tan Z., Song W., Li R. (2026). Design Principle of Adhesive Hydrogels for Biomedical Application. Mater. Horiz..

[6] Chen Y., Guo X., Zhang Y., Yang Z., Meng J., Li P., Ni Y., Huang Z., Wu H., Wei Q. (2026). Recent Advances in Plant Polyphenol-Based Adhesive Hydrogels for Biomedical Applications. Biomater. Adv..

[7] Kasai R.D., Radhika D., Archana S., Shanavaz H., Koutavarapu R., Lee D.-Y., Shim J. (2023). A Review on Hydrogels Classification and Recent Developments in Biomedical Applications. Int. J. Polym. Mater. Polym. Biomater..

[8] Petraglia F.M., Giordano S., Santoro A. (2026). Functional Hydrogels in Bone Tissue Engineering: From Material Design to Translational Applications. Biologics.

[9] Mansuri A., Gupta D., Pawar R., Tanwar S.S. (2025). A Comprehensive Review of Hydrogel Classification, Fabrication, and Utility. Int. J. Res. Publ. Rev..

[10] Silva I., Khalil N., Fan C.L.F., Wang Y.E., Leveridge B., Miceli G.C., Lee A., Goding J., Green R.A., Boshier P.R. (2026). Hydrogel-Based Drug Delivery Systems for Localised Treatment of Peritoneal Metastasis: A Systematic and IDEAL Framework Review. Crit. Rev. Oncol./Hematol..

[11] Verma A., Devi P., Bhogal S., Jasrotia R., Thakur V.K. (2026). Lignin-Based Hydrogels for Sustainable Agriculture: Extraction, Design, and Applications. ACS Environ. Au.

[12] Zubair M., Batool F., Muzammil S., Azeem F., Rasul I., Afzal M., Siddique M.H. (2026). Emerging Trends of Graphene Based Hydrogels in Agriculture Applications. Graphene Hydrogels.

[13] Li N., Ma Z., Feng W., Zhu Y., Zhu X., Wang H., Wang F. (2026). Holistic Utilization of Lignin-Derived Oligomers and Polymers from Oxidative Catalysis to Fabricate Highly Swelling Agro-Hydrogels. Green Chem..

[14] Li X., Xu R., Xie C., Ge Z., Gao B., Lim C.T. (2026). Microscale Architectures for Intelligent Soft Robotics: From Functional Microneedles to Biointegrated Wearable Systems. Nano-Micro Lett..

[15] Akram T., Zhang B., Zhao G. (2026). MXene-Polymer Hydrogel Sensors for Next-Generation Advanced Wearable Sensing: From Synthesis to Real World Integration. Adv. Mater. Technol..

[16] He X., Wei Y., Xu K. (2025). Hydrogel-Based Treatment of Diabetic Wounds: From Smart Responsive to Smart Monitoring. Gels.

[17] Zhao L.-L., Shi X.-L., Huang C.-H., Zou M.-L., Liang M.-Y., Liang J., Kang S.-M., Miao L., Chen Z.-G. (2025). Temperature-Sensitive Ionic Hydrogels for Dual Electric and Thermal Responsive Smart Window. Chem. Eng. J..

[18] Zhu T., Ni Y., Biesold G.M., Cheng Y., Ge M., Li H., Huang J., Lin Z., Lai Y. (2023). Recent Advances in Conductive Hydrogels: Classifications, Properties, and Applications. Chem. Soc. Rev..

[19] Chen L., Liu F., Abdiryim T., Liu X. (2024). Stimuli-Responsive Hydrogels as Promising Platforms for Soft Actuators. Mater. Today Phys..

[20] Xu J., Zou Y., Chen H., Wan Z., Takagi A., Wang Z., Yu J., Liu L., Lu Y., Fan Y. (2025). Magnetoresponsive Cellulose Nanofiber Hydrogels: Dynamic Structuring, Selective Light Transmission, and Information Encoding. ACS Nano.

[21] Yu Z., Gu Y., Ren Y., Li Z., Mou C., Wu Z., Wu D., Mou J. (2024). Smart Hydrogels for Shape Deformation: Mechanism, Preparation, and Properties. J. Mater. Chem. C.

[22] Guo W.-Y., Jiang Y.-T., Wang B., Ma M.-G. (2026). Multifunctional Cellulose Nanofiber/MXene Zwitterionic Hydrogel for Dual-Mode Strain and Temperature Sensing with High-Performance Electromagnetic Shielding. J. Mater. Sci. Technol..

[23] Saha H., Dey B., Hossain K.R. (2025). Hydrogel-Based Biosensors in Biomedical Applications. Biomater. Connect.

[24] Fatimi A., Damiri F., El Arrach N., Hemdani H., Musuc A.M., Berrada M. (2025). Hydrogel-Based Biomaterials: A Patent Landscape on Innovation Trends and Patterns. Gels.

[25] El-Husseiny H.M., Mady E.A., Hamabe L., Abugomaa A., Shimada K., Yoshida T., Tanaka T., Yokoi A., Elbadawy M., Tanaka R. (2022). Smart/Stimuli-Responsive Hydrogels: Cutting-Edge Platforms for Tissue Engineering and Other Biomedical Applications. Mater. Today Bio.

[26] Song X., Dong X., Liu H., Wang Z., Cao Q. (2026). Applications of Stimuli-Responsive Hydrogels in Renewable Energy: A Review. ChemSusChem.

[27] Kumar A., Gupta R. (2023). Hydrogels: Fundamentals to Advanced Energy Applications.

[28] García-Torres J., Alemán C., Gupta R.K. (2024). Multifunctional Hydrogels: From Basic Concepts to Advanced Applications.

[29] Tang Y., Petropoulos K., Kurth F., Gao H., Migliorelli D., Guenat O., Generelli S. (2020). Screen-Printed Glucose Sensors Modified with Cellulose Nanocrystals (CNCs) for Cell Culture Monitoring. Biosensors.

[30] Sheraz M., Sun X.-F., Siddiqui A., Wang Y., Hu S., Sun R. (2025). Cellulose-Based Electrochemical Sensors. Sensors.

[31] Wang S., Zhang C., Ma J., Zhang L., Wang Z. (2025). Dual-Functionalization Cellulose Nanofiller Use to Simultaneously Enhance Hydrogel Sensors’ Mechanical and Electrical Properties. ACS Appl. Polym. Mater..

[32] Kim H.J., Koo J.H., Lee S., Hyeon T., Kim D.-H. (2025). Materials Design and Integration Strategies for Soft Bioelectronics in Digital Healthcare. Nat. Rev. Mater..

[33] Chen R., Guo R., Li Y., Yan H., Huang J., Xu H., Shi W., Zhang Z., Zhuo S., Liu M. (2026). Hydrogel Ionic Sensory Systems. Mater. Chem. Front..

[34] Oh S., Lee S., Kim S.W., Kim C.Y., Jeong E.Y., Lee J., Kwon D.A., Jeong J.-W. (2024). Softening Implantable Bioelectronics: Material Designs, Applications, and Future Directions. Biosens. Bioelectron..

[35] Choi Y., Jin P., Lee S., Song Y., Tay R.Y., Kim G., Yoo J., Han H., Yeom J., Cho J.H. (2025). All-Printed Chip-Less Wearable Neuromorphic System for Multimodal Physicochemical Health Monitoring. Nat. Commun..

[36] Lee M.Y., Lee E.S., Ko N.Y., Kim H.J., Kim D.-H., Cha G.D., Koo J.H. (2025). Emerging Roles of Hydrogels, Organogels, and Their Hybrids in Soft Bioelectronics and Bioplatforms. npj Biosens..

[37] Lee H.K., Yang Y.J., Koirala G.R., Oh S., Kim T. (2024). From Lab to Wearables: Innovations in Multifunctional Hydrogel Chemistry for next-Generation Bioelectronic Devices. Biomaterials.

[38] Yu Y., Liang X., Ruan H., Wang T., Li Y., Wen Z. (2025). Hydrogel-Based Sensors for Multimodal Health Monitoring: From Material Design to Intelligent Sensing. Nanoscale.

[39] Sun X., Agate S., Salem K.S., Lucia L., Pal L. (2020). Hydrogel-Based Sensor Networks: Compositions, Properties, and Applications—A Review. ACS Appl. Bio Mater..

[40] Mo F., Lin Y., Liu Y., Zhou P., Yang J., Ji Z., Wang Y. (2025). Advances in Ionic Conductive Hydrogels for Skin Sensor Applications. Mater. Sci. Eng. R Rep..

[41] Li L., Sun X., Guo Y., Cheng W., Shi Y., Pan L. (2025). Recent Advances in Stimuli-Responsive Conductive Hydrogels for Smart Sensing and Actuation: Properties, Design Strategies, and Applications. Macromol. Mater. Eng..

[42] Su Y., Zhang Y., Zhao Z., Du Z., Lei C., Yang Q., Liu N., Bai R., Zhu L., Xu W. (2026). Advanced Hydrogel Sensing Platforms: Rational Design, Signal Transduction Mechanisms, and Multidimensional Applications. Coord. Chem. Rev..

[43] Du T., Cui T., Yang B., Wu B., Zhang C., Shang K., Yang Y., Wu J., Gao Y., Wang M. (2026). Multifunctional Flexible Sensor Based on P (AA-NIPAM)/PVA Dual-Network Hydrogel for Monitoring Soccer Players’ Motion. Adv. Mater. Technol..

[44] Ahmed I., Sakr A., Samad Y.A., Butt H. (2025). Advanced Hydrogel Optical Fiber Sensors with Triple-Readout for Real-Time pH Sensing. Sci. Rep..

[45] Zhou J., Zheng J., Wang C., Fan M., Wang S., Xiong F., Li Y., Yang C. (2026). Fabrication of High-Toughness PEDOT: PSS-Based Conductive Hydrogel Strain/Temperature Sensors. RSC Adv..

[46] Luo Q., Wei C., Zhou Y., Chen W., Wen C., Qing N., Lu Z., Tang L. (2026). Antiswelling Charge-Transfer Hydrogels for Highly Sensitive Underwater Temperature Sensing. ACS Appl. Polym. Mater..

[47] Wu K., Jiao M., Yang M., Qiao Y., He Q., Fei T., Yang Z. (2025). Dual-Mode, Self-Powered, and Flexible Humidity Sensor Based on Double-Network Hydrogel with Multifunctional Applications. Small Methods.

[48] Li Y., Wen X., Li X., Zahid M., Wang H., Zhang J. (2025). Design of Super Stretchability, Rapid Self-Healing, and Self-Adhesion Hydrogel Based on Starch for Wearable Strain Sensors. Carbohydr. Polym..

[49] Mo F., Zhou P., Lin S., Zhong J., Wang Y. (2024). A Review of Conductive Hydrogel-based Wearable Temperature Sensors. Adv. Healthc. Mater..

[50] Du X., Zhai J., Li X., Zhang Y., Li N., Xie X. (2021). Hydrogel-Based Optical Ion Sensors: Principles and Challenges for Point-of-Care Testing and Environmental Monitoring. ACS Sens..

[51] Aham E.C., Ravikumar A., Zeng K., Arunjegan A., Tamilselvan G., Hu Z., Zhang Z., Zhao H. (2025). Intelligent Hydrogel and Smartphone-Assisted Colorimetric Sensor Based on Bi-Metallic Organic Frameworks for Effective Detection of Kanamycin and Oxytetracycline. Microchim. Acta.

[52] EL-Sharif H.F., Stevenson D., Warriner K., Reddy S.M. (2014). Hydrogel-Based Molecularly Imprinted Polymers for Biological Detection.

[53] Qi M., Han Y., Zhang W., Liu Y., Jiang D., Wu Z., Xu M., Fu J., Li B. (2025). Advances and Future Perspectives in Hydrogel-Based Sensing Technologies: A Comprehensive Review. Nanotechnology.

[54] Li W., Xu C., Li J., Liu Y., Chen Z., Liu H., Yin Y., Zhai W., Zhou K., Dai K. (2025). Highly Stretchable, Adhesive and Self-Healing Hydrogel Electronics for Human Motion Detection and Electrophysiological Signal Monitoring. J. Colloid Interface Sci..

[55] Chen Y., Fan H., Liu W., Wang J., Wang T., Yang R., Zhang L., Shang L., Wen D. (2026). Wearable Microneedle Patch Integrated with Metal Hydrogel-Based Signal Probe for Dermal Interstitial Fluid Protein Biomarkers Monitoring. Adv. Mater..

[56] Niu Y., Zhao Z., Yang L., Lv D., Sun R., Zhang T., Li Y., Bao Q., Zhang M., Wang L. (2025). Towards Intelligent Wound Care: Hydrogel-Based Wearable Monitoring and Therapeutic Platforms. Polymers.

[57] Aroche A.F., Nissan H.E., Daniele M.A. (2025). Hydrogel-forming Microneedles and Applications in Interstitial Fluid Diagnostic Devices. Adv. Healthc. Mater..

[58] Zhuo S., Tessier A., Arefi M., Zhang A., Williams C., Ameri S.K. (2024). Reusable Free-Standing Hydrogel Electronic Tattoo Sensors with Superior Performance. npj Flex. Electron..

[59] Wu Z., Qiao Z., Chen S., Fan S., Liu Y., Qi J., Lim C.T. (2024). Interstitial Fluid-Based Wearable Biosensors for Minimally Invasive Healthcare and Biomedical Applications. Commun. Mater..

[60] Völlmecke K., Afroz R., Bierbach S., Brenker L.J., Frücht S., Glass A., Giebelhaus R., Hoppe A., Kanemaru K., Lazarek M. (2022). Hydrogel-Based Biosensors. Gels.

[61] Liu J., Gao X., Ma W., Zhang L., Wen X., Zhai M., Chai G., Fan W., Zhang Q., Wei R. (2026). Flexible Hydrogel Sensor Based on MoS_2_ for Highly Selective Dopamine Detection against Catecholamine Cross-Interference. Analyst.

[62] Fang K., Wan Y., Wei J., Chen T. (2023). Hydrogel-Based Sensors for Human–Machine Interaction. Langmuir.

[63] Zhang M., Xu T., Liu K., Zhu L., Miao C., Chen T., Gao M., Wang J., Si C. (2025). Modulation and Mechanisms of Cellulose-based Hydrogels for Flexible Sensors. SusMat.

[64] Liang X., Chen S., Liang Y., Wang M., Wang Q., Chen D., Ma X., Ding H., Zhong H.-J. (2026). Alginate-Based Hydrogels: Recent Progress in Preparation, Property Tuning, and Multifunctional Applications. Gels.

[65] Xu J., Lv S., Chen Y., Liu X., He T., Liu L. (2026). A Review on Chitosan-Based Hydrogels with Conductive and Stimulus-Responsive Properties for Smart Sensing and Food Packaging. Microchem. J..

[66] Gong J., Ma X., Ding J., Huang X., Zhu C., Wei Z., Yuan J., Ao Y., Yuan B. (2026). Highly Thermoelectric Chitosan-Based Hydrogel for Integrated Thermal Safety Protection System: Cooling, Overheating Warning, and Thermal Runaway Suppression in Lithium-Ion Battery. Int. J. Biol. Macromol..

[67] Song X., Guo J., Zhang Y., Liu M., Chen W., Ji X., Guan F. (2026). High-Strength Polyacrylamide/Sodium Alginate-Based Hydrogel with Extreme Environmental Tolerance for Low-Temperature Strain Sensing. Colloids Surf. A Physicochem. Eng. Asp..

[68] Gu B., Wang S., Zhou A., Wu X., Zhang Q., Huang B., Lin B., Xu C., Wei Y., Fu L. (2026). A Polyacrylamide/Gelatin Hydrogel with Superior Durability and Weather Resistance for Sustainable Wearable Electronics. Polymer.

[69] Zhang Q., Zhai G., Zhang X., Li S., Wei Y., Xing Z., Wang Z.-X. (2026). Improved Polyacrylamide/Chitosan Dual-Network Hydrogels through the Synergistic Effect of Aluminum Ions and Imidazolium Ionic Liquids toward Flexible Capacitors and Sensors. Polymer.

[70] Zúniga M.M.G., Oh E., Nguyen T.B., Ding R., Duan Z., Nam J.-D., Suhr J. (2026). Chemical-Responsive Ortho-Vanillin Functionalized PEG Hydrogel as Colorimetric Sensor for Biogenic Amines Detection in Smart Food Packaging Materials. Food Packag. Shelf Life.

[71] Zou L., Li Y., Feng S., Wang Z., Xiao H., Chen S., Wang Y., He L., Mao X. (2025). Innovations and Applications of Composite Hydrogels: From Polymer-Based Systems to Metal-Ion-Doped and Functional Nanomaterial-Enhanced Architectures. Small.

[72] Manchi P., Paranjape M.V., Kurakula A., Kavarthapu V.S., Kim C.-W., Yu J.S. (2025). Graphene Oxide-Incorporated PVA/Sodium Alginate Composite Hydrogel-Based Flexible and Sensitive Single-Electrode TENGs for Efficient Energy Harvesting and Smart Security Applications. Nano Energy.

[73] Li W., Wang F., Liu J., Wang J., Deng L. (2025). Super-Adhesive and Highly Sensitive Conductive Hydrogel Based on Halometallate Ionic Liquid for Flexible Electronic Devices. Chem. Eng. J..

[74] Muhammad U., Cao X., Zhang T., Ji W., Lv R., Chen J., Wei Y. (2025). Fabrication of Highly Tough, Self-Healing Sodium Alginate/Polyacrylamide and Copper Based Nanocomposite Hydrogel and Its Application as Strain and Pressure Sensor for Human Health Monitoring and Signature Recognition. Int. J. Biol. Macromol..

[75] Amara U., Xu L., Hussain I., Yang K., Hu H., Ho D. (2025). MXene Hydrogels for Soft Multifunctional Sensing: A Synthesis-centric Review. Small.

[76] Han M., Luo D., Talha K., He J., Xing M., Chen L., Liu H. (2025). Research Progress On Conductive Hydrogels and Their Applications in Flexible Sensors: A Review. J. Mater. Chem. A.

[77] Zhang Y., Wu B.M. (2023). Current Advances in Stimuli-Responsive Hydrogels as Smart Drug Delivery Carriers. Gels.

[78] Protsak I.S., Morozov Y.M. (2025). Fundamentals and Advances in Stimuli-Responsive Hydrogels and Their Applications: A Review. Gels.

[79] Roy A., Manna K., Pal S. (2022). Recent Advances in Various Stimuli-Responsive Hydrogels: From Synthetic Designs to Emerging Healthcare Applications. Mater. Chem. Front..

[80] Zhang D., Ren B., Zhang Y., Xu L., Huang Q., He Y., Li X., Wu J., Yang J., Chen Q. (2020). From Design to Applications of Stimuli-Responsive Hydrogel Strain Sensors. J. Mater. Chem. B.

[81] Cui C., Fu Q., Meng L., Hao S., Dai R., Yang J. (2020). Recent Progress in Natural Biopolymers Conductive Hydrogels for Flexible Wearable Sensors and Energy Devices: Materials, Structures, and Performance. ACS Appl. Bio Mater..

[82] Lim H.-R., Kim H.S., Qazi R., Kwon Y.-T., Jeong J.-W., Yeo W.-H. (2020). Advanced Soft Materials, Sensor Integrations, and Applications of Wearable Flexible Hybrid Electronics in Healthcare, Energy, and Environment. Adv. Mater..

[83] Song H., Jung D.H., Cho Y., Cho H.H., Panferov V.G., Liu J., Heo J.H., Lee J.H. (2025). Nanoparticle-Integrated Hydrogels as Versatile Colorimetric Sensors. Coord. Chem. Rev..

[84] Tavakoli J., Tang Y. (2017). Hydrogel Based Sensors for Biomedical Applications: An Updated Review. Polymers.

[85] Chenani H., Saeidi M., Rastkhiz M.A., Bolghanabadi N., Aghaii A.H., Orouji M., Hatamie A., Simchi A. (2024). Challenges and Advances of Hydrogel-Based Wearable Electrochemical Biosensors for Real-Time Monitoring of Biofluids: From Lab to Market. A Review. Anal. Chem..

[86] Wang Z., Li N., Yang X., Zhang Z., Zhang H., Cui X. (2024). Thermogalvanic Hydrogel-Based e-Skin for Self-Powered on-Body Dual-Modal Temperature and Strain Sensing. Microsyst. Nanoeng..

[87] Pham X.-H., Shim S., Kim T.-H., Hahm E., Kim H.-M., Rho W.-Y., Jeong D.H., Lee Y.-S., Jun B.-H. (2017). Glucose Detection Using 4-Mercaptophenyl Boronic Acid-Incorporated Silver Nanoparticles-Embedded Silica-Coated Graphene Oxide as a SERS Substrate. BioChip J..

[88] Theyagarajan K., Saikrithika S., Kim Y.-J. (2026). MXene- and MOF-Based Hydrogels: Emerging Platforms for Electrochemical Biosensing and Health Monitoring. Micromachines.

[89] Chen Z., Liu S., Yu W., Wang L., Lv F., Yang L., Yu H., Shi H., Huang Y. (2025). Hydrogel Based Flexible Wearable Sweat Sensor for SERS-AI Monitoring Treatment Effect of Lung Cancer. Sens. Actuators B Chem..

[90] Nanda D., Behera D., Pattnaik S.S., Behera A.K. (2025). Advances in Natural Polymer-Based Hydrogels: Synthesis, Applications, and Future Directions in Biomedical and Environmental Fields. Discov. Polym..

[91] Irimia-Vladu M., Sariciftci N.S. (2025). Natural Polymers for Emerging Technological Applications: Cellulose, Lignin, Shellac and Silk. Polym. Int..

[92] Gusain R., Chaudhary U., Rana V., Joshi G., Gupta P.K., Bachheti R.K., Worku L.A. (2026). Transforming Cellulose for Sustainability: Comprehensive Insights into Modification Approaches and Their Applications. ACS Omega.

[93] Nascimento D.M., Nunes Y.L., Figueirêdo M.C.B., de Azeredo H.M.C., Aouada F.A., Feitosa J.P.A., Rosa M.F., Dufresne A. (2018). Nanocellulose Nanocomposite Hydrogels: Technological and Environmental Issues. Green Chem..

[94] Madhushree M., Mahesha G.T., Venkatachalam H., Bhat K.S. (2026). Green Approaches to the Surface Modification of Cellulose: Methods and Mechanisms. J. Compos. Sci..

[95] Salem K.S., Starkey H.R., Pal L., Lucia L., Jameel H. (2019). The Topochemistry of Cellulose Nanofibrils as a Function of Mechanical Generation Energy. ACS Sustain. Chem. Eng..

[96] Jing X., Li H., Mi H.-Y., Liu Y.-J., Feng P.-Y., Tan Y.-M., Turng L.-S. (2019). Highly Transparent, Stretchable, and Rapid Self-Healing Polyvinyl Alcohol/Cellulose Nanofibril Hydrogel Sensors for Sensitive Pressure Sensing and Human Motion Detection. Sens. Actuators B Chem..

[97] Mirshafiei M., Afshar A.K., Yazdian F., Rashedi H., Rahdar A., Ali Aboudzadeh M. (2026). Bacterial Cellulose: A Sustainable Nanostructured Polymer for Biosensor Development. RSC Sustain..

[98] Wang X., Cheng J., Han Z., Cheng W., Han G., Wang Y., Wang D. (2026). Superelastic and Highly Sensitive Conductive Hydrogel Sensor Enabled by Spatially Confined Assembly of MXene within Bacterial Cellulose Network. Int. J. Biol. Macromol..

[99] Wei J., Liu C., Shi L., Liu Y., Lu H. (2025). High-Performance Conductive Double-Network Hydrogel Base on Sodium Carboxymethyl Cellulose for Multifunctional Wearable Sensors. Carbohydr. Polym..

[100] Wang H., Song R., Chen G., Wang F., Wang L., Lei J., Liu J. (2026). Biomass-Based Dual-Network Multifunctional Hydrogel for Durable Wearable Sensors and Emergency Hemostasis. Chem. Eng. J..

[101] Xie Y., Chen L., Liu F., Jing X., Li Y., Su M., Abdiryim T., Liu X. (2025). Multifunctional Ionic Conductive Hydrogels Based on Sodium Carboxymethyl Cellulose and Poly (Ionic Liquid) for High-Performance Supercapacitors and Sensing Applications. Polymer.

[102] Tomić S.L., Babić Radić M.M., Vuković J.S., Filipović V.V., Nikodinovic-Runic J., Vukomanović M. (2023). Alginate-Based Hydrogels and Scaffolds for Biomedical Applications. Mar. Drugs.

[103] Ren Y., Wang Q., Xu W., Yang M., Guo W., He S., Liu W. (2024). Alginate-Based Hydrogels Mediated Biomedical Applications: A Review. Int. J. Biol. Macromol..

[104] Sun Z., Dong C., Chen B., Li W., Hu H., Zhou J., Li C., Huang Z. (2023). Strong, Tough, and Anti-Swelling Supramolecular Conductive Hydrogels for Amphibious Motion Sensors. Small.

[105] Bao Z., Xian C., Yuan Q., Liu G., Wu J. (2019). Natural Polymer-Based Hydrogels with Enhanced Mechanical Performances: Preparation, Structure, and Property. Adv. Healthc. Mater..

[106] Chen H., Huang J., Liu J., Gu J., Zhu J., Huang B., Bai J., Guo J., Yang X., Guan L. (2021). High Toughness Multifunctional Organic Hydrogels for Flexible Strain and Temperature Sensor. J. Mater. Chem. A.

[107] Xu X., Xu Q., Ma J., Deng Y., An W., Yan K., Zong Y., Zhang F. (2024). Progress in Protein-Based Hydrogels for Flexible Sensors: Insights from Casein. ACS Sens..

[108] Ling Q., Fan X., Ling M., Liu J., Zhao L., Gu H. (2023). Collagen-Based Organohydrogel Strain Sensor with Self-Healing and Adhesive Properties for Detecting Human Motion. ACS Appl. Mater. Interfaces.

[109] Liu J., Li S., Li S., Tian J., Li H., Pan Z., Lu L., Mao Y. (2024). Recent Advances in Natural-Polymer-Based Hydrogels for Body Movement and Biomedical Monitoring. Biosensors.

[110] Demeter M., Călina I., Scărișoreanu A., Micutz M. (2021). E-Beam Cross-Linking of Complex Hydrogels Formulation: The Influence of Poly(Ethylene Oxide) Concentration on the Hydrogel Properties. Gels.

[111] Li J., Shi S., Wang W., Bai L., Chen H., Wei K., Yang L. (2025). Seawater-Assisting Antifreezing Hydrogels with Functional Cellulose Nanocrystals for Wearable Flexible Sensors. Chem. Eng. J..

[112] Li G., Gao Y., Sun C., Niu F., Shi Z., Yang Q., Xiong C. (2024). An Anti-Freezing and Anti-Drying Nanocellulose Hydrogel for Human Motion Detection. Colloids Surf. A Physicochem. Eng. Asp..

[113] Cui D., Sun Y., Li T., Hu Z., Zhao Y., Wang X., Chen S., Toktarbay Z., Wei H. (2025). Mechanically Robust Polyacrylamide/Gelatin Ionic Hydrogels Reinforced by Sodium Alginate for Wearable Device Applications. Adv. Compos. Hybrid Mater..

[114] Zhao K., Zhao X., Guo X., Zhao Y., Tong M., Chen L., Zhuang S., Gu X. (2026). Switchable, Recyclable, and Time-Programmable Hydrogels via Counterion-Engineered Differential Metal-Ion Coordination. Adv. Funct. Mater..

[115] Fu H., Wang B., Li J., Xu J., Li J., Zeng J., Gao W., Chen K. (2022). A Self-Healing, Recyclable and Conductive Gelatin/Nanofibrillated Cellulose/Fe^3+^ Hydrogel Based on Multi-Dynamic Interactions for a Multifunctional Strain Sensor. Mater. Horiz..

[116] Yan R., Sun Q., Shi X., Sun Z., Tan S., Tang B., Chen W., Liang F., Yu H.-D., Huang W. (2023). Skin-Interfaced Self-Powered Pressure and Strain Sensors Based on Fish Gelatin-Based Hydrogel for Wireless Wound Strain and Human Motion Detection. Nano Energy.

[117] Li Z., Chen L., Liu F., Liu X. (2025). Chitosan-Based Hydrogels with Stretchable, Self-Healing, Self-Adhesive Properties for Flexible Sensing Applications. Colloids Surf. A Physicochem. Eng. Asp..

[118] Huo H., Shi H., Yang H., Zhang X., Wan J., Shen J., Du G., Yang L. (2024). A Conductive Hydrogel with Excellent Self-Adhesion, Sensitivity, and Stability for Wearable Strain Sensors to Monitor Human Motion. J. Mater. Chem. A.

[119] Li X., Li X., Yan M., Wang Q. (2023). Chitosan-Based Transparent and Conductive Hydrogel with Highly Stretchable, Adhesive and Self-Healing as Skin-like Sensor. Int. J. Biol. Macromol..

[120] Lu L., Huang Z., Li X., Li X., Cui B., Yuan C., Guo L., Liu P., Dai Q. (2022). A High-Conductive, Anti-Freezing, Antibacterial and Anti-Swelling Starch-Based Physical Hydrogel for Multifunctional Flexible Wearable Sensors. Int. J. Biol. Macromol..

[121] Ma C., Xie F., Wei L., Zheng C., Liu X., Wang L., Liu P. (2022). All-Starch-Based Hydrogel for Flexible Electronics: Strain-Sensitive Batteries and Self-Powered Sensors. ACS Sustain. Chem. Eng..

[122] Tai Y.-T., Wei C.-Y., Ko F.-H. (2025). Hydrogel-Based Colorimetric Power-Saving Sensors for on-Site Detection of Chloride Ions and Glucose in Sweat. Biosens. Bioelectron..

[123] Liu X., Shi H., Song F., Yang W., Yang B., Ding D., Liu Z., Hui L., Zhang F. (2024). A Highly Sensitive and Anti-Freezing Conductive Strain Sensor Based on Polypyrrole/Cellulose Nanofiber Crosslinked Polyvinyl Alcohol Hydrogel for Human Motion Detection. Int. J. Biol. Macromol..

[124] Wang Y., Yao A., Dou B., Huang C., Yang L., Liang J., Lan J., Lin S. (2024). Self-Healing, Environmentally Stable and Adhesive Hydrogel Sensor with Conductive Cellulose Nanocrystals for Motion Monitoring and Character Recognition. Carbohydr. Polym..

[125] Sun Z., Yu B., Dong C., Yu C., Sheng L., Cui Z., Liu Y., Lu Z., Chen B., Xie D. (2026). Dual-Mode Sensor with Saturated Mechanochromic Structural Color Enhanced by Black Conductive Hydrogel for Interactive Rehabilitation Monitoring. Nano-Micro Lett..

[126] Quan Z., Chen Z., Li H., Sun S., Xu Y. (2024). A Hydrogel Sensor Based on Cellulose Nanofiber/Polyvinyl Alcohol with Colorimetric-Fluorescent Bimodality for Non-Invasive Detection of Urea in Sweat. Int. J. Biol. Macromol..

[127] Wang X., Zeng M., Torrens M., Niu P., Fernandez Sanchez C., Gich M., Roig A. (2025). Eco-Friendly Conformal and Self-Adhesive Electrochemical Sensors for Sweat Monitoring. ACS Appl. Mater. Interfaces.

[128] Segneanu A.-E., Bejenaru L.E., Bejenaru C., Blendea A., Mogoşanu G.D., Biţă A., Boia E.R. (2025). Advancements in Hydrogels: A Comprehensive Review of Natural and Synthetic Innovations for Biomedical Applications. Polymers.

[129] Song Y., Han J., Wang B., Li S., Wang X., Zhang J., Li N., Sheng X., Shi H., Shao C. (2026). Tough and Self-Adhesive Nanolignin Multifunctional Hydrogel-Based Strain Sensor for HumanMachine Interaction. ChemSusChem.

[130] Li X., Wang Y., Tian Y., Zhang L., Ma J. (2025). Biomimetic Multiscale Structure with Hierarchically Entangled Topologies of Cellulose-Based Hydrogel Sensors for Human-Computer Interaction. Carbohydr. Polym..

[131] Zhu L., Pan Y., Wu J., Du Z., Shao Z.-B. (2023). Polyelectrolyte Microgels-Enhanced Double-Crosslinking Polyacrylamide Hydrogel Sensing with Stretchable, Transparent, and Fast Response. Eur. Polym. J..

[132] Das A., Babu A., Chakraborty S., Van Guyse J.F., Hoogenboom R., Maji S. (2024). Poly(*N*-isopropylacrylamide) and Its Copolymers: A Review on Recent Advances in the Areas of Sensing and Biosensing. Adv. Funct. Mater..

[133] Mao Y., Gao M., Qian C., Zhang N., Miao R., Fan X., Li Y. (2026). Multi-Responsive and Self-Sensing Flexible Actuators Based on Conductive Polypyrrole/Poly(*N*-Isopropylacrylamide) Hydrogels. Sens. Actuators A Phys..

[134] Luan K., Chen R., Qu M., Si Z., Bai H., Shi X. (2026). Preparation and Characterization of Silica Nanoparticle–Reinforced PAA/PEG Dual-Network Hydrogels with High Compressive Strength after Swelling. Colloid Polym. Sci..

[135] Wang Z., Wei H., Huang Y., Wei Y., Chen J. (2023). Naturally Sourced Hydrogels: Emerging Fundamental Materials for next-Generation Healthcare Sensing. Chem. Soc. Rev..

[136] Afshar H., Kamran F., Moshiri H., Shahi F. (2026). Soft and Stretchable Conductive Polymers for Next-Generation Wearable Biosensing. Polym. Eng. Sci..

[137] Yu X., Wang Y., Zhang H., Li Z., Zheng Y., Fan X., Lv Y., Zhang X., Liu T. (2023). Strain-Stiffening, Self-Healing, and Low-Hysteresis Physically Dual-Cross-Linked Hydrogels Derived from Two Mechanically Distinct Hydrogen Bonds. Chem. Mater..

[138] Wang K., Li M., Hu J., Cheng Y., Kong Y., Li A. (2026). Anti-Freezing Conductive PAA Hydrogel Based on Tannic Acid-Modified MWCNT for Flexible Strain Sensors. J. Mater. Sci. Mater. Electron..

[139] He Y., Xiong J., Hu Y., Guo Z., Wang S., Mao J. (2025). AM/AMPS Delignified Wood-Based Hydrogel with Enhanced Mechanical Strength and Fatigue Resistance for Wearable Strain Sensing and Energy Harvesting. Polymer.

[140] Bibi M., Asif A., Ali I., Hassan G., Shuja A., Shahzada S., Fahad S., Murtaza I., Parraman C. (2025). Self-Healing and Ultra-Stretchable Hydrogel Strain Sensor Based on Poly (AAm-co-AMPS) for Multifunctional Biomedical Applications. Adv. Sens. Res..

[141] Roy A., Zenker S., Jain S., Afshari R., Oz Y., Zheng Y., Annabi N. (2024). A Highly Stretchable, Conductive, and Transparent Bioadhesive Hydrogel as a Flexible Sensor for Enhanced Real-time Human Health Monitoring. Adv. Mater..

[142] Zhang H., Yue M., Wang T., Wang J., Wu X., Yang S. (2021). Conductive Hydrogel-Based Flexible Strain Sensors with Superior Chemical Stability and Stretchability for Mechanical Sensing in Corrosive Solvents. New J. Chem..

[143] Zhang D., Tang Y., Zhang Y., Yang F., Liu Y., Wang X., Yang J., Gong X., Zheng J. (2020). Highly Stretchable, Self-Adhesive, Biocompatible, Conductive Hydrogels as Fully Polymeric Strain Sensors. J. Mater. Chem. A.

[144] Jin X., Jiang H., Qiao F., Huang W., Bao X., Wang Z., Hu Q. (2021). Fabrication of alginate-P (SBMA-co-AAm) Hydrogels with Ultrastretchability, Strain Sensitivity, Self-adhesiveness, Biocompatibility, and Self-cleaning Function for Strain Sensors. J. Appl. Polym. Sci..

[145] Wu Z., Yang X., Wu J. (2021). Conductive Hydrogel- and Organohydrogel-Based Stretchable Sensors. ACS Appl. Mater. Interfaces.

[146] Priya A.S., Premanand R., Ragupathi I., Bhaviripudi V.R., Aepuru R., Kannan K., Shanmugaraj K. (2024). Comprehensive Review of Hydrogel Synthesis, Characterization, and Emerging Applications. J. Compos. Sci..

[147] Zhou P., Zhang Z., Mo F., Wang Y. (2024). A Review of Functional Hydrogels for Flexible Chemical Sensors. Adv. Sens. Res..

[148] Zhang J., Wang Z. (2022). Nanoparticle–Hydrogel Based Sensors: Synthesis and Applications. Catalysts.

[149] Zhang Y., Li X., Wu W., Hong W., Jiao T. (2026). 3D Printed Hydrogel Flexible Sensors: Fabrication Techniques, Sensing Mechanisms, and Application Advances. ACS Appl. Polym. Mater..

[150] Mogli G., Chiappone A., Sacco A., Pirri C.F., Stassi S. (2023). Ultrasensitive Piezoresistive and Piezocapacitive Cellulose-Based Ionic Hydrogels for Wearable Multifunctional Sensing. ACS Appl. Electron. Mater..

[151] Zhang Y.X., He Y., Liang Y., Tang J., Yang Y., Song H.M., Zrínyi M., Chen Y.M. (2023). Sensitive Piezoresistive Pressure Sensor Based on Micropyramid Patterned Tough Hydrogel. Appl. Surf. Sci..

[152] Uysal B., Madduma-Bandarage U.S., Jayasinghe H.G., Madihally S. (2025). 3D-Printed Hydrogels from Natural Polymers for Biomedical Applications: Conventional Fabrication Methods, Current Developments, Advantages, and Challenges. Gels.

[153] Zhang M., Hakobyan K., Cheng C., Xu J. (2025). Surface Engineering of Polymer Hydrogels toward Functional Soft Material Innovations. Macromol. Chem. Phys..

[154] Lv H., Zong S., Li T., Zhao Q., Xu Z., Duan J. (2023). Room Temperature Ca^2+^-Initiated Free Radical Polymerization for the Preparation of Conductive, Adhesive, Anti-Freezing and UV-Blocking Hydrogels for Monitoring Human Movement. ACS Omega.

[155] Hu J., Shan F., Tian Y., Wei J., Chen Z., Liu W., Chen G., Fu G. (2025). Deep Eutectic Solvent-Mediated Sunlight Polymerization for Rapid Fabrication of Degradable Hydrogel-Based Wearable Sensors. Chem. Eng. J..

[156] Tordi P., Tamayo A., Jeong Y., Han B., Al Kayal T., Cavallo A., Bonini M., Samorì P. (2026). Fully Bio-Based Gelatin Organohydrogels via Enzymatic Crosslinking for Sustainable Soft Strain and Temperature Sensing. Adv. Funct. Mater..

[157] Li Z., Lu F., Liu Y. (2023). A Review of the Mechanism, Properties, and Applications of Hydrogels Prepared by Enzymatic Cross-Linking. J. Agric. Food Chem..

[158] Zhang A., Huang H., Shen J., Feng X., Duan L., Wang J., Zhang X. (2025). A Synergistic “Pre-Crosslinking-Freeze Thawing-Salting out-Coordination” Tactic to Design Antimicrobial and Highly Conductive PVA/PEI Hydrogels with Excellent Mechanical Performance. Chem. Eng. J..

[159] Sun Y., Xie Y., Zou H., Chen Y., Wen Z., Liang Q., Peng X., Sui J., Chen J., He Y. (2024). Fabrication and Application of Multifunctional Conductive Hydrogel Film for Wearable Sensors via Efficient Freeze-Thaw Cycling and Annealing Process. Chem. Eng. J..

[160] Zhang J., Gu X., Chang F., Lv Z., Ma N., Zhu X., Zhang X. (2025). The Anti-Swelling, Freezing Resistant and High Strength PVA Organohydrogel Combined with Multifunctional {Mo154} for Sensitive and Wide-Range Strain Sensing. Eur. Polym. J..

[161] Huang C., Zhong Y., Cai W., Cao L., Wang Q., Li W., Lin Z., Zhang P. (2025). Highly Sensitive, Anti-Freeze, and Ion-Conductive Polyelectrolyte-Based Hydrogel for Flexible Sensor Applications in Sub-Zero Temperatures. Polymer.

[162] Huang X., Zhang L. (2024). Encapsulation of Hydrogel Sensors. Chem. Eng. J..

[163] Wang C., Ding Y., Wu T., Li Z., Hu C., Wang Z., Zhou Y., Lin X., Zhang W., Xu J. (2025). Ionic Double-network Hydrogels for Integrated Electromagnetic Shielding and Self-powered Sensing in Wearable Electronics. Adv. Sci..

[164] Wang Y., Gao Y., Tang L., Guo Y., Sha B., Jiang Y. (2026). Hydrogel-Based Wearable and Implantable Biosensors in Health Monitoring. Biomater. Sci..

[165] Jiang Y., Zhan D., Zhang M., Zhu Y., Zhong H., Wu Y., Tan Q., Dong X., Zhang D., Hadjichristidis N. (2023). Strong and Ultra-tough Ionic Hydrogel Based on Hyperbranched Macro-cross-linker: Influence of Topological Structure on Properties. Angew. Chem..

[166] Jaseem S.A., Rahmani P., Sakorikar T., Ma J., Almutairi O., Voinov M.A., Smirnov A.I., Chen B., Dickey M.D. (2026). Liquid Metals as Initiators of Free-Radical Polymerization of Hydrogels: A Perspective. Adv. Funct. Mater..

[167] Di X., Wang Y., Yu B., Ran W., Zhang R., Gao X., Yuan C. (2026). A Highly Resilient, Conductive, and Anti-Swelling Hybrid-Crosslinked Hydrogel Based on a Semi-Interpenetrating Network for Multimodal Sensing and Marine Monitoring. J. Mater. Chem. A.

[168] Li Y., Zhu J., Chen L., Chen N., Chen X., Lv J. (2025). Polysaccharide-Driven Self-Healing Dual-Network Hydrogel via Schiff Base for High-Performance Flexible Sensing. Carbohydr. Polym..

[169] Wan Z., Zhang H., Niu M., Guo Y., Li H. (2025). Self-Healing Conductive Hydrogel Based on a Schiff Base Bond for Digital Light Processing 3D Printing. ACS Appl. Polym. Mater..

[170] Carrascal-Hernández D.C., Grande-Tovar C.D., Insuasty D., Márquez E., Mendez-Lopez M. (2026). Versatility of Click Chemistry in Hydrogel Synthesis: From Molecular Strategies to Applications in Regenerative Medicine. Gels.

[171] Atmani Z. (2025). Development of Tailored Polysaccharide Gels Using Click Chemistry Approaches. Ph.D. Thesis.

[172] Jin S., Oh D.X., Park J. (2026). Dynamic Disulfide Chemistry for Functional Polymers: Self-Healing, Vitrimer Behavior, and Biochemical/Electronic Applications. ChemSusChem.

[173] Shi W., Ching Y.C., Mo F., Chuah C.H. (2026). Recent Advances in Flexible Hybrid Hydrogel-Based Sensors for Human Health Monitoring. Small.

[174] Hasturk O., Jordan K.E., Choi J., Kaplan D.L. (2020). Enzymatically Crosslinked Silk and Silk-Gelatin Hydrogels with Tunable Gelation Kinetics, Mechanical Properties and Bioactivity for Cell Culture and Encapsulation. Biomaterials.

[175] Wang L., Peng S., Patil A., Jiang J., Zhang Y., Chang C. (2022). Enzymatic Crosslinked Silk Fibroin Hydrogel for Biodegradable Electronic Skin and Pulse Waveform Measurements. Biomacromolecules.

[176] Hsiao L.-Y., Jing L., Li K., Yang H., Li Y., Chen P.-Y. (2020). Carbon Nanotube-Integrated Conductive Hydrogels as Multifunctional Robotic Skin. Carbon.

[177] Sarkar S.K., Takei K. (2025). Toward Environmentally Friendly Hydrogel-Based Flexible Intelligent Sensor Systems. Adv. Intell. Discov..

[178] Luo R., Zhang K., Li H., Cui Y., Hu J., Zhou X., Wang W., Li L. (2025). Highly Robust Conductive Hydrogel Based on In-Situ Polymerization of PEDOT for Wearable Devices. Appl. Mater. Today.

[179] Medha, Sethi S., Behal K., Thakur S., Sharma N., Kumar A., Kaur A., Kaith B.S. (2026). Dual Function Polysaccharide Based Hybrid Hydrogel for Drug Delivery and Fluorescence Sensing. J. Polym. Res..

[180] Li S., Pei M., Wan T., Yang H., Gu S., Tao Y., Liu X., Zhou Y., Xu W., Xiao P. (2020). Self-Healing Hyaluronic Acid Hydrogels Based on Dynamic Schiff Base Linkages as Biomaterials. Carbohydr. Polym..

[181] Ding X., Zhang L., Jiang C., Liu S., Li H., Xi J., Wu D. (2024). Building Covalent Crosslinks of Carboxymethyl Konjac Glucomannan with Boronic Ester Bonds for Fabricating Multimodal Hydrogel Sensor. Int. J. Biol. Macromol..

[182] Sawicki L.A., Kloxin A.M. (2014). Design of Thiol–Ene Photoclick Hydrogels Using Facile Techniques for Cell Culture Applications. Biomater. Sci..

[183] Malafaia A.P., Sobreiro-Almeida R., Rodrigues J.M., Mano J.F. (2025). Thiol-Ene Click Chemistry: Enabling 3D Printing of Natural-Based Inks for Biomedical Applications. Biomater. Adv..

[184] Hutomo D.I., Deandra F.A., Ketherin K., García-Gareta E., Bachtiar E.W., Amir L., Tadjoedin F.M., Widaryono A., Haerani N., Lessang R. (2024). The Effect of Carbodiimide Crosslinkers on Gelatin Hydrogel as a Potential Biomaterial for Gingival Tissue Regeneration. Gels.

[185] Chung K.-Y., Halwachs K.N., Lu P., Sun K., Silva H.A., Rosales A.M., Page Z.A. (2022). Rapid Hydrogel Formation via Tandem Visible Light Photouncaging and Bioorthogonal Ligation. Cell Rep. Phys. Sci..

[186] Xia S., Zhang Q., Song S., Duan L., Gao G. (2019). Bioinspired Dynamic Cross-Linking Hydrogel Sensors with Skin-like Strain and Pressure Sensing Behaviors. Chem. Mater..

[187] Li Y., Liu R., Li Y., Chen G., Wan Y., Tian Y. (2026). PVA-Based Composite Hydrogels for Biomedical Applications. J. Mater. Chem. C.

[188] Charlet A., Lutz-Bueno V., Mezzenga R., Amstad E. (2021). Shape Retaining Self-Healing Metal-Coordinated Hydrogels. Nanoscale.

[189] Wang R., Kim S.H., Sun F., Zheng X., Jiang F., Wang X., Diao B., Zhang H., Li X., Li R. (2025). Bio-Inspired Hydrogen Bonding Cross-Linking Strategy for DIW-Printed Carbon-Based Conductive Hydrogels in Wearable Self-Powered Sensing Systems. ACS Appl. Electron. Mater..

[190] Cao X., Cao Q., Zhang T., Ji W., Muhammad U., Chen J., Wei Y. (2024). Hydrophobically Associated Hydrogel for High Sensitivity and Resolution of an Interdigital Electrode Pressure Sensor. Biomacromolecules.

[191] Yazdani S., Khan M., Shahzad A., Shah L.A., Ye D. (2023). Ionic Conductive Hydrogels Formed through Hydrophobic Association for Flexible Strain Sensing. Sens. Actuators A Phys..

[192] Moussa M., El-Kady M.F., Dubal D., Tung T.T., Nine M.J., Mohamed N., Kaner R.B., Losic D. (2020). Self-Assembly and Cross-Linking of Conducting Polymers into 3D Hydrogel Electrodes for Supercapacitor Applications. ACS Appl. Energy Mater..

[193] Ding Y., Hou Y., Liu W., Liu Y., Liu Q., Yao Y., Chen D. (2025). Wearable Strain Sensor Based on Double Network PVA/SA/CNT Hydrogel for Reliable Electrocardiography and Motion Monitoring. Sens. Actuators A Phys..

[194] Wang X., Li N., Yin J., Wang X., Xu L., Jiao T., Qin Z. (2023). Interface Interaction-Mediated Design of Tough and Conductive MXene-Composited Polymer Hydrogel with High Stretchability and Low Hysteresis for High-Performance Multiple Sensing. Sci. China Mater..

[195] Chen Y., Zhang D., Wang Z., Tang M., Zhang H. (2024). Pb^2+^ Transfer-Enabled Recoverable Hydrogel-Based H_2_S Colorimetric Sensing with Assistance of Multimodal Deep Learning for Multifunctional Applications. Adv. Funct. Mater..

[196] Madhuvilakku R., Jeong O.C., Hong Y. (2025). Bimetallic Pd@ CeO_2_-Decorated 2D-g-C_3_N_4_/Calcium Alginate Conductive Hydrogel: A Multifunctional Hybrid Material for Wearable Electrochemical Sensing of Serotonin. Adv. Compos. Hybrid Mater..

[197] Wen Y., Li X., Zhang S., Xie C., Ma W., Liang L., He Z., Duan H., Mou Y., Zhao G. (2022). Preparation of a “Branch-Fruit” Structure Chitosan Nanofiber Physical Hydrogels with High Mechanical Strength and pH-Responsive Controlled Drug Release Properties. RSC Adv..

[198] Liu X., Liu J., Lin S., Zhao X. (2020). Hydrogel Machines. Mater. Today.

[199] Cherifi K., Matoori S. (2025). Hydrogels for Analyte Sensing. ACS Meas. Sci. Au.

[200] Erfkamp J., Guenther M., Gerlach G. (2019). Piezoresistive Hydrogel-Based Sensors for the Detection of Ammonia. Sensors.

[201] Erfkamp J., Guenther M., Gerlach G. (2018). Hydrogel-Based Piezoresistive Sensor for the Detection of Ethanol. J. Sens. Sens. Syst..

[202] Chen Y., Guo S., Teng K., An Q. (2026). Piezoionics: Strategies and Applications for Mechanical-to-Ionic Transduction. Nano Res..

[203] Chen Y., Lv C., Ye X., Ping J., Ying Y., Lan L. (2025). Hydrogel-Based Pressure Sensors for Electronic Skin Systems. Matter.

[204] Park K., Yuk H., Yang M., Cho J., Lee H., Kim J. (2022). A Biomimetic Elastomeric Robot Skin Using Electrical Impedance and Acoustic Tomography for Tactile Sensing. Sci. Robot..

[205] Zhou S., Zhang Y.-J., Zhang C., Yi C.-X., Han T.-H., Huang J.-H., Ding L., Peng Y.-R., Sun T.-S. (2024). Multidimensional Biomimetic Construction of Highly Sensitive Wrinkled Gels Based on the Transformation of Multiple Supramolecular Interactions Induced by Dual-Solvent Strategy. Adv. Funct. Mater..

[206] Mo F., Huang Y., Li Q., Wang Z., Jiang R., Gai W., Zhi C. (2021). A Highly Stable and Durable Capacitive Strain Sensor Based on Dynamically Super-tough Hydro/Organo-gels. Adv. Funct. Mater..

[207] Chang Y., Wang L., Li R., Zhang Z., Wang Q., Yang J., Guo C.F., Pan T. (2021). First Decade of Interfacial Iontronic Sensing: From Droplet Sensors to Artificial Skins. Adv. Mater..

[208] Mirzaee M., Askari-Sedeh M., Zolfagharian A., Baghani M. (2025). Hydrogel-Based Capacitive Sensor Model for Ammonium Monitoring in Aquaculture. Adv. Eng. Mater..

[209] Chen M., Ghorbanzadeh S., Zhang W. (2025). A Low-Energy-Dissipating Hydrogel-Based Capacitive Sensor for Therapeutic Pressure Monitoring. Sens. Actuators A Phys..

[210] Bai C., Dong X., Liu Q., Zhao M., Yang K., Niu Y., Zhang H., Zhai W. (2026). A Self-Powered Hydrogel Electronic Skin with Decoupled Multimodal Sensing for Closed-Loop Human-Machine Interactions. Nat. Commun..

[211] Song Y., Xia Y., Zhang W., Yu Y., Cui Y., Liu L., Zhang T., Liu S., Zhao H., Fei T. (2024). Humidity-Activated Ammonia Sensor Based on Carboxylic Functionalized Cross-Linked Hydrogel. Sensors.

[212] Wu Z., Wang H., Ding Q., Tao K., Shi W., Liu C., Chen J., Wu J. (2023). A Self-Powered, Rechargeable, and Wearable Hydrogel Patch for Wireless Gas Detection with Extraordinary Performance. Adv. Funct. Mater..

[213] Dhanjai, Sinha A., Kalambate P.K., Mugo S.M., Kamau P., Chen J., Jain R. (2019). Polymer Hydrogel Interfaces in Electrochemical Sensing Strategies: A Review. TrAC Trends Anal. Chem..

[214] Yun J., Kang J., Sharipov M., Ryu W. (2025). Recent Progress in Hydrogel Microneedle Sensors Based on Electrochemical and Optical Sensing. Appl. Spectrosc. Rev..

[215] Golshahi H., Dashtian K., Zare-Dorabei R., Kerman K. (2026). Recent Progress in Wearable Electrochemical Sensors Based on MXene-Conductive Hydrogels. Analyst.

[216] Yao M., Hsieh J.-C., Wang H. (2026). Hydrogel-Integrated Multimodal Physiological and Modulation Systems. Mater. Horiz..

[217] Chen L., Chang X., Chen J., Zhu Y. (2022). Ultrastretchable, Antifreezing, and High-Performance Strain Sensor Based on a Muscle-Inspired Anisotropic Conductive Hydrogel for Human Motion Monitoring and Wireless Transmission. ACS Appl. Mater. Interfaces.

[218] Yang X., Wang P., Wu X., Liao Y., Liu S., Duan W., Yue Y. (2024). Exploring the Mechanism, Advancements, and Application of Thermogalvanic Effect in Hydrogels. Chin. J. Chem..

[219] Hu Q., Wu H., Yu C., Xiao H., Shen C., Zhao Z., Yao Y., Yong X., Liang X., Wu H. (2026). A Machine Learning-Enabled Planar Interdigitated Thermogalvanic Hydrogel for Synergistic Thermal and Strain Sensing. J. Mater. Chem. A.

[220] Saeidi M., Chenani H., Orouji M., Adel Rastkhiz M., Bolghanabadi N., Vakili S., Mohamadnia Z., Hatamie A., Simchi A. (2023). Electrochemical Wearable Biosensors and Bioelectronic Devices Based on Hydrogels: Mechanical Properties and Electrochemical Behavior. Biosensors.

[221] Mei C., Li L., Zhang J., Pan L., Yang F., Wang Z., Yang L. (2023). Ion-Induced Enhanced Fluorescence Colorimetric Hydrogel Sensor for Visual Quantization of Doxycycline. Sens. Actuators B Chem..

[222] Li F., Huang W., Xie S., Xin L., Lu X., Li Z., Zhang X., Li X., Chen W. (2026). A Piezoionic Hydrogel-Based Electrochemical Strain Sensor for Self-Powered Pulse Monitoring and Machine Learning-Assisted Speech Recognition. ACS Sens..

[223] Cheng N., Luo Q., Yang Y., Shao N., Nie T., Deng X., Chen J., Zhang S., Huang Y., Hu K. (2025). Injectable pH Responsive Conductive Hydrogel for Intelligent Delivery of Metformin and Exosomes to Enhance Cardiac Repair after Myocardial Ischemia-Reperfusion Injury. Adv. Sci..

[224] Xing L., Song Y., Zou X., Tan H., Yan J., Wang J. (2023). A Mussel-Inspired Semi-Interpenetrating Structure Hydrogel with Superior Stretchability, Self-Adhesive Properties, and pH Sensitivity for Smart Wearable Electronics. J. Mater. Chem. C.

[225] Maag H. (2007). Prodrugs of Carboxylic Acids. Prodrugs. Biotechnology: Pharmaceutical Aspects.

[226] Asgharian H., Kammarchedu V., Gu S.J., Ebrahimi A. (2025). WoundMx: Multiplexed Detection of Wound Infection Biomarkers with a Multimodal Sensor System Based on Laser-Induced Graphene. npj 2D Mater. Appl..

[227] Kiti K., Sirikarn P., Konkayan A., Chuysinuan P., Suwantong O. (2025). Potential Use of Hydrogels Based on Alginate, Carboxymethyl Cellulose, and Poly(Vinyl Alcohol) Containing Amoxicillin/Butterfly Pea Flower Extract for pH-Responsive Antibacterial Wound Dressings. Int. J. Biol. Macromol..

[228] Albay M.M., Abbasiasl T., Oral C.B., Beker L. (2025). Methacrylated Chitosan Methacrylated Poly(Vinyl Alcohol)-Based Hydrogel Patch for Long-Term Electrochemical Wound pH Sensing. ACS Sens..

[229] Zhu Z., Xie Q., Sun Y., Yan F., Kang X., Zalewski P., Gou X. (2025). Electrically Controlled On-Demand Wound Therapy Based on Real-Time pH Monitoring. Chem. Eng. J..

[230] Odinotski S., Dhingra K., GhavamiNejad A., Zheng H., GhavamiNejad P., Gaouda H., Mohammadrezaei D., Poudineh M. (2022). A Conductive Hydrogel-Based Microneedle Platform for Real-Time pH Measurement in Live Animals. Small.

[231] Güngör Z., Ozay H. (2022). Ultra-Fast pH Determination with a New Colorimetric pH-Sensing Hydrogel for Biomedical and Environmental Applications. React. Funct. Polym..

[232] Wang W., Wu C., Zhu K., Chen F., Zhou J., Shi Y., Zhang C., Li R., Wu M., Zhuo S. (2021). Real-Time Personal Fever Alert Monitoring by Wearable Detector Based on Thermoresponsive Hydrogel. ACS Appl. Polym. Mater..

[233] Yang G., Xie B., Tian J., Zhang Y., Liu Y., Gong H., Pang B., Qiu Y., Gong C., Bu T. (2026). A Wireless Hydrogel Thermotherapy System with Adhesion-Customizable Interfaces for Accelerating Wound Healing. Adv. Mater..

[234] Wang P., Wang G., Sun G., Bao C., Li Y., Meng C., Yao Z. (2025). A Flexible-Integrated Multimodal Hydrogel-Based Sensing Patch. Nano-Micro Lett..

[235] Jiang J., Tian Y., Wu X., Zeng M., Wu C., Wei D., Luo H., Sun J., Ding J., Fan H. (2025). Temperature and Light Dual-Responsive Hydrogels for Anti-Inflammation and Wound Repair Monitoring. J. Mater. Chem. B.

[236] Liu H., Chu H., Yuan H., Li D., Deng W., Fu Z., Liu R., Liu Y., Han Y., Wang Y. (2024). Bioinspired Multifunctional Self-Sensing Actuated Gradient Hydrogel for Soft-Hard Robot Remote Interaction. Nano-Micro Lett..

[237] Hasallari F., Gallo E., Rizzuti S., Diaferia C., Salvatore M., Accardo A., Gianolio E., Aime S. (2025). A Biocompatible, Highly Sensitive Hydrogel-Based T1 Thermometer for in Vivo MRI Applications. Mater. Today Chem..

[238] Li M., Pu J., Cao Q., Zhao W., Gao Y., Meng T., Chen J., Guan C. (2024). Recent Advances in Hydrogel-Based Flexible Strain Sensors for Harsh Environment Applications. Chem. Sci..

[239] Xia S., Song S., Jia F., Gao G. (2019). A Flexible, Adhesive and Self-Healable Hydrogel-Based Wearable Strain Sensor for Human Motion and Physiological Signal Monitoring. J. Mater. Chem. B.

[240] Rahmani P., Shojaei A. (2021). A Review on the Features, Performance and Potential Applications of Hydrogel-Based Wearable Strain/Pressure Sensors. Adv. Colloid Interface Sci..

[241] Fan Z., Ji D., Kim J. (2023). Recent Progress in Mechanically Robust and Conductive-hydrogel-based Sensors. Adv. Intell. Syst..

[242] Sun X., Yao F., Li J. (2020). Nanocomposite Hydrogel-Based Strain and Pressure Sensors: A Review. J. Mater. Chem. A.

[243] Zhou J., Shao Y., Song X., Chen T., Lu X. (2026). High-Performance Polydopamine and Silver Nanoparticle-Modified MXene-Based Hydrogel Flexible Strain Sensors for Transfer-Learning-Assisted Handwriting Recognition and Wrist-Movement Monitoring. Nanoscale.

[244] Liu P., Zhang H., Gao L., Li Y., Gu Y., Li X., Liu Y., Wang Z., Zou D. (2025). High-Strength, Moisturizing and Conductive Hydrogels for Flexible Strain Sensors via Photoinitiated Polymerization and Ion-Solvent Displacement. J. Polym. Sci..

[245] Liu Q., Xie M., Wang C., Deng M., Li P., Yang X., Zhao N., Huang C., Zhang X. (2024). Rapid Preparation Triggered by Visible Light for Tough Hydrogel Sensors with Low Hysteresis and High Elasticity: Mechanism, Use and Recycle-by-Design. Small.

[246] Yuan W., Wang F., Qu X., Wang S., Lei B., Shao J., Wang Q., Lin J., Wang W., Dong X. (2023). In Situ Rapid Synthesis of Hydrogels Based on a Redox Initiator and Persistent Free Radicals. Nanoscale Adv..

[247] Zhang X., Sun H., Zhang J., Wang Z. (2025). A Highly Sensitive and Stable Mxene/Bacterial Cellulose Double Network Hydrogel Flexible Strain Sensor for Human Activities Monitoring. J. Appl. Polym. Sci..

[248] Ji Q., Li Y., Wang Z., Tan X., Sun L., Li S., Wang C., Chen R., Chu F., Nan J. (2025). A Highly Tough and Strain-Sensitive MXene Hydrogel Sensor Enabling Integrated Wearable Electronics with Body Conformability and Real-Time Visualization. Small.

[249] Zeng R., Lu S., Qi C., Jin L., Xu J., Dong Z., Lei C. (2022). Polyacrylamide/Carboxymethyl Chitosan Double-Network Hydrogels with High Conductivity and Mechanical Toughness for Flexible Sensors. J. Appl. Polym. Sci..

[250] Liu Y., Liu R., Liu H., Li D., Fu S., Jin K., Cheng Y., Fu Z., Xing F., Tian Y. (2024). Tough, High Conductivity Pectin Polysaccharide-Based Hydrogel for Strain Sensing and Real-Time Information Transmission. Int. J. Biol. Macromol..

[251] Yuan T., Li C., Georgopoulou A., Kolinski J.M., Amstad E. (2026). Hydrogel-Based 3D-Printable Stretchable Pressure Sensor. Adv. Mater. Technol..

[252] Zhou T., Li P., Sun Y., Wang W., Bai L., Chen H., Yang H., Yang L., Wei D. (2025). BSA/PEI/GOD Modified Cellulose Nanocrystals for Construction of Hydrogel-Based Flexible Glucose Sensors for Sweat Detection. J. Mater. Chem. B.

[253] Xia F., Chen J., Yang P., Shi M., Wang B., Zhang D. (2026). A Biocompatible Microsensor Based on a Cation-Selective Nanoporous Conductive Hydrogel for Stable Electrochemical Sensing of Dopamine in Mouse Brain. ACS Sens..

[254] Qu Y., Chen R., Chen J., Li Q., Abukhadra M.R., ElSherbeeny A.M., Jin L., Jiang Q., Feng S., Bian S. (2025). Hydrogel-Based Electrochemical Microneedles Biosensor for Sensitive Monitoring of Lactic Acid in Interstitial Fluid. Anal. Chim. Acta.

[255] Nie Z., Qu X., He M., Zhen S., Peng K., Chen Y. (2025). Hydrogels for Emerging Wearable Sweat Monitoring Devices. Intell. Sports Health.

[256] Lin P.-H., Sheu S.-C., Chen C.-W., Huang S.-C., Li B.-R. (2022). Wearable Hydrogel Patch with Noninvasive, Electrochemical Glucose Sensor for Natural Sweat Detection. Talanta.

[257] Yuan Q., Fang H., Wu X., Wu J., Luo X., Peng R., Xu S., Yan S. (2023). Self-Adhesive, Biocompatible, Wearable Microfluidics with Erasable Liquid Metal Plasmonic Hotspots for Glucose Detection in Sweat. ACS Appl. Mater. Interfaces.

[258] He Y., Gu F., Guo Z. (2025). Multi-Response Hydrogel for Real-Time Monitoring of Changes in Environmental Factors. Chem. Eng. J..

[259] Hosseinlou R., Dargahi M., Vanashi A.K. (2024). Alkaline Range pH Sensor Based on Chitosan Hydrogel: A Novel Approach to pH Sensing. Int. J. Biol. Macromol..

[260] Wu R., Wang Z., Fu Y., Jiang J., Chen Y.-C., Liu T. (2025). High-Sensitive Hydrogel Optofluidic Microcavities for Heavy Metal Ion Detection. ACS Sens..

[261] Mia R., Tuli M.B., Hossen M.A., Rimi S.A., Rahman S., Raju M.R.H., Mithi N.J., Hossain K.R. (2025). Hydrogel-Based Nanocomposites for Enhanced Environmental Remediation. Environ. Funct. Mater..

[262] Yenduri S., Naga Prashant K. (2026). Application of Hydrogels in Heavy Metal Sensing in Wastewater. Applications of Hydrogels in Modern Wastewater Treatment.

[263] Nunekpeku X., Li H., Zahid A., Li C., Zhang W. (2025). Advances in Hydrogel-Integrated SERS Platforms: Innovations, Applications, Challenges, and Future Prospects in Food Safety Detection. Biosensors.

[264] Wang Z., Sheng W., Tang X., Ya T., Jin Z., Wang S., Ji Q., Fan C., Liu Y. (2025). Temperature-Sensitive Driving Assembled Fluorescence Hydrogel Based Dual-Mode Sensor for Adsorbing and Detecting of Heavy Metal Cadmium Ions in Food and Water. Food Chem..

[265] Chen H., Zhan X.-Q., Hong Z.-L., Ni W., Zhu C.-C., Leng Y.-P., Chen C.-C., Guo R.-T., Ma N., Tsai F.-C. (2024). Hydrogel-Based Sensor for the Detection of Iron Ions and Excessive Alerting. Langmuir.

[266] He W., Wu Z., Liu X., Xu H., Jian X., Zhou K., Du J., Wang J., Zhang D. (2025). MOF-Based Fluorescent Hydrogels for Rapid and Highly Selective Visual Detection of Heavy Metal Ions. Microchem. J..

[267] Yang J., Luo Z., Wang M. (2022). Novel Fluorescent Nanocellulose Hydrogel Based on Nanocellulose and Carbon Dots for Detection and Removal of Heavy Metal Ions in Water. Foods.

[268] Guo J., Huang H., Zhou M., Yang C., Kong L. (2018). Quantum Dots-Doped Tapered Hydrogel Waveguide for Ratiometric Sensing of Metal Ions. Anal. Chem..

[269] Peng Z., Yu H.-R., Wen J.-Y., Wang Y.-L., Liang T., Cheng C.-J. (2022). A Novel Ion-Responsive Photonic Hydrogel Sensor for Portable Visual Detection and Timely Removal of Lead Ions in Water. Mater. Adv..

[270] Mao X., Mao D., Jiang J., Su B., Chen G., Zhu X. (2021). A Semi-Dry Chemistry Hydrogel-Based Smart Biosensing Platform for on-Site Detection of Metal Ions. Lab A Chip.

[271] Wang T., Wu L., Zhang C., Zhang G., Chen Y., Gao C., Wang Z., Wang Y., Gao Y., Xuan F. (2025). Humidity-Compatible Chemiresistive Hydrogel Sensor for Real-Time Breath CO_2_ Monitoring. Chem. Eng. J..

[272] Wu S., Yang Y., Cheng Y., Wang S., Zhou Z., Zhang P., Zhu X., Wang B., Zhang H., Xie S. (2023). Fluorogenic Detection of Mercury Ion in Aqueous Environment Using Hydrogel-based AIE Sensing Films. Aggregate.

[273] Chu Q., Liu Z., Feng F., Chen D., Qin J., Bai Y., Feng Y. (2024). A Novel Bio-Based Fluorescent N, P-CDs@ CMC/PEI Composite Hydrogel for Sensitive Detection and Efficient Capture of Toxic Heavy Metal Ions. J. Hazard. Mater..

[274] Du X., Zhai J., Zeng D., Chen F., Xie X. (2020). Distance-Based Detection of Calcium Ions with Hydrogels Entrapping Exhaustive Ion-Selective Nanoparticles. Sens. Actuators B Chem..

[275] Nille O.S., Kolekar A.G., Devre P.V., Koparde S.V., Sawat A.H., Sohn D., Patole S.P., Anbhule P.V., Gore A.H., Kolekar G.B. (2025). Nanocarbon Eco-Hydrogel Kit: On-Site Visual Metal Ion Sensing and Dye Cleanup, Advancing the Circular Economy in Environmental Remediation. Analyst.

[276] Ren Y., Chen M., Zhang L., Pan R., Wu H., Yang W. (2026). CO_2_ Responsive Hydrogel-Based High Sensitivity Pb^2+^ Optical Fiber Sensor with Green and in Situ Desorption. Talanta.

[277] Wu J., Wu Z., Xu H., Wu Q., Liu C., Yang B.-R., Gui X., Xie X., Tao K., Shen Y. (2019). An Intrinsically Stretchable Humidity Sensor Based on Anti-Drying, Self-Healing and Transparent Organohydrogels. Mater. Horiz..

[278] Luo Y., Li J., Ding Q., Wang H., Liu C., Wu J. (2023). Functionalized Hydrogel-Based Wearable Gas and Humidity Sensors. Nano-Micro Lett..

[279] Jung C., Kim S.-J., Jang J., Ko J.H., Kim D., Ko B., Song Y.M., Hong S.-H., Rho J. (2022). Disordered-Nanoparticle–Based Etalon for Ultrafast Humidity-Responsive Colorimetric Sensors and Anti-Counterfeiting Displays. Sci. Adv..

[280] Cui Y., Du Y., Jia Y., Xu W., Bi Y., Xia X., Yang Q. (2026). Fast and High-Response Humidity Sensor Based on Ionically Crosslinked Gum Tragacanth Hydrogel. Sens. Actuators B Chem..

[281] Ham M., Kim S., Lee W., Lee H. (2022). Fabrication of Printable Colorimetric Food Sensor Based on Hydrogel for Low-Concentration Detection of Ammonia. Biosensors.

[282] Du X., Gu S., Wang X., Zhang S., Zhang B., Yu G., Wang Z., Chen W., Li Q. (2025). The Preparation of SiO_2_/GO/PVA Based Hydrogel Sensor and Its Application for Rapid and Sensitive Detection of NH_3_. Sens. Actuators B Chem..

[283] Zhi H., Gao J., Feng L. (2020). Hydrogel-Based Gas Sensors for NO_2_ and NH_3_. ACS Sens..

[284] Jia Z., Zhang J., Fu H., Ji Z., Zhang J., Yang X., Shi C., Sun X., Guo Y. (2025). Flexible Fluorescent Sensor Based on a Heterojunction-Functionalized Hydrogel with Dual-Mode Crosslinking for H_2_S Monitoring during Fish Spoilage. Chem. Eng. J..

[285] Wang S., Gerlach G., Körner J. (2023). A Study of Smart Hydrogels as Sensing Elements in Gaseous Environment for VOC Detection. Polymer.

[286] Wang K., Zhang J., Li H., Wu J., Wan Q., Chen T., Liu W., Peng H., Zhang H., Luo Y. (2024). Smart Hydrogel Sensors for Health Monitoring and Early Warning. Adv. Sens. Res..

[287] Zhang C., Kang Y., Li G., Zhou J., Yao C., Zeng H., Wu C., Wang J. (2025). Multifunctional Integration of Hydrogel-Based Sensors and Their Applications in Fire Early Warning Systems. J. Polym. Sci..

[288] Ding H., Wei Y., Wu Z., Tao K., Ding M., Xie X., Wu J. (2020). Recent Advances in Gas and Humidity Sensors Based on 3D Structured and Porous Graphene and Its Derivatives. ACS Mater. Lett..

[289] Wu J., Tao K., Miao J., Norford L.K. (2015). Improved Selectivity and Sensitivity of Gas Sensing Using a 3D Reduced Graphene Oxide Hydrogel with an Integrated Microheater. ACS Appl. Mater. Interfaces.

[290] Ding Q., Wang H., Zhou Y., Zhang Z., Luo Y., Wu Z., Yang L., Xie R., Yang B.-R., Tao K. (2025). Self-Powered Switchable Gas-Humidity Difunctional Flexible Chemosensors Based on Smart Adaptable Hydrogel. Adv. Mater..

